# Mental health, academic performance, and addictive behaviors among university student carers

**DOI:** 10.3389/fpsyg.2026.1971038

**Published:** 2026-10-14

**Authors:** Giulia Landi, Kenneth I. Pakenham, Federica Ambrosini, Zhangxuan Bao, Eliana Tossani, Silvana Grandi

**Affiliations:** 1 Department of Psychology “Renzo Canestrari”, University of Bologna, Bologna, Italy; 2 Laboratory of Psychosomatics and Clinimetrics, Department of Psychology “Renzo Canestrari”, University of Bologna, Cesena, Italy; 3 School of Psychology, The University of Queensland, Brisbane, QLD, Australia; 4 Department of Education Sciences “G. M. Bertin”, University of Bologna, Bologna, Italy

**Keywords:** academic performance, addictive behaviors, caregiving, mental health, student carers, university students

## Abstract

**Objectives:**

Student mental health is an emerging global public health priority in higher education. Preliminary evidence indicates that caregiving is a mental health risk factor in university students. This study investigates demographic, educational, and caregiving variables as potential risk factors for university student carer mental health (anxiety, depression), addictive behaviors, and academic performance problems.

**Methods:**

2,533 university students in Italy completed an online cross-sectional survey assessing depressive symptoms (Patient Health Questionnaire-9), anxiety symptoms (Generalized Anxiety Disorder-7), 10 addictive behaviors (Screener for Substance and Behavioral Addictions), and self-assessed academic performance. Predictors included demographics variables (sex, age, nationality), educational variables (degree programme, residential status, enrollment status), caregiving context variables (carer-care recipient relationship, care recipient's type of health condition) and caregiving responsibilities.

**Results:**

Student carers living with a family member with a serious health condition comprised 30.1% of the sample (*n* = 762). Compared with non-carers, carers reported higher depressive symptoms (*B* = 1.83, 95% CI [1.33, 2.33]; β = 0.14), higher anxiety symptoms (*B* = 1.32, 95% CI [0.87, 1.77]; β = 0.11), and lower academic performance (*B* = −0.21, 95% CI [−0.39, −0.02]; β = −0.05). Carer status was also associated with higher odds of problematic involvement in cannabis (OR = 1.69, 95% CI [1.25, 2.28]), food consumption (OR = 1.62, 95% CI [1.34, 1.95]), shopping (OR = 1.39, 95% CI [1.09, 1.78]), videogaming (OR = 1.29, 95% CI [1.06, 1.57]), and social-network use (OR = 1.26, 95% CI [1.06, 1.50]). Within the student-carer subgroup, after adjusting for all demographic, educational, caregiving-context, and caregiving-responsibility variables, caring for a family member with a mental health condition or intellectual disability was associated with higher depressive symptoms (*B* = 1.66, 95% CI [0.54, 2.79]). Greater caregiving responsibilities were associated with higher depressive symptoms (*B* = 1.12, 95% CI [0.62, 1.62]), higher anxiety symptoms (*B* = 1.01, 95% CI [0.57, 1.44]), and higher odds of problematic food consumption (OR = 1.32, 95% CI [1.10, 1.59]), and physical exercise (OR = 1.31, 95% CI [1.08, 1.59]).

**Conclusion:**

Findings replicate prior evidence showing student carers are at higher risk than non-carer peers for mental health problems. Results extend prior research showing student carers are also at higher risk for addictive behaviors and poorer self-assessed academic performance. Findings highlight the incremental and simultaneous contributions of demographic, educational, and caregiving variables as risk factors for student carer mental health and addictive behaviors.

## 1 Introduction

An emerging public health priority confronting the higher education sector is the widely documented increase in mental health problems among university students (Lipson et al., 2019). Identifying university students at greater risk for mental health problems is essential in the pursuit of targeted prevention endeavors. Caregiving has been identified as a mental health risk factor in adolescents, young adults and adults (Bowman Grangel et al., 2025; Lacey et al., 2022; Landi et al., 2022d; Methi et al., 2024). Preliminary evidence suggests that caregiving is also a mental health risk factor among university students as they balance the demands of caregiving and higher education (Armstrong-Carter et al., 2022; Haugland et al., 2020). However, recent reviews indicate a dearth of research into university student carers, particularly with respect to identifying factors associated with increased risk of mental health problems (Knopf et al., 2022; Runacres et al., 2024).

Student carers are university students who undertake unpaid caregiving support for a family member who has a serious health condition including, but not limited to, physical and/or mental illness or disability. Student carers undertake a diverse range of caregiving activities, including practical help (e.g., preparing meals), personal care, administering medication, support with financial matters and physical help (Knopf et al., 2022; Haugland et al., 2022). Through these activities, student carers contribute to informal family care while pursuing their university studies. The literature on youth caregiving conceptualizes contributions to family functioning along a continuum, ranging from routine household assistance to extensive caregiving responsibilities and activities that may constrain developmental opportunities (Pakenham and Cox, 2015; Landi et al., 2022c). Caregiving responsibilities refer to the psychological sense of duty or responsibility related to undertaking these care tasks as part of the caregiving role involved in contributing to family functioning (Pakenham and Cox, 2015; Landi et al., 2025). University student carers providing unpaid care to a family member with a serious health condition constitute a critical component of the healthcare workforce.

Overall, few studies have investigated university student carers providing care to a family member with a serious health condition. A systematic review (Knopf et al., 2022) and a wider scoping review (Runacres et al., 2024) of university student carer research identified 6 and 14 eligible studies, respectively. Both reviews found evidence suggesting caregiving responsibilities have adverse effects on student carer mental health and university performance. For example, compared to non-carer peers, young adult student carers have been found to report higher depression, anxiety and stress (Haugland et al., 2020; Greene et al., 2017). A mental health dimension neglected by prior university student carer research is addictive behaviors. One study that has addressed this issue found that Thai student carers were more likely to engage in risky health behaviors such as smoking and drinking, and they were more likely to have a higher body mass index compared to non-carers (Yiengprugsawan et al., 2012).

The adverse impact of caregiving on university performance is a common theme identified in the university student carer research. Findings from qualitative studies show that student carers report challenges in reconciling the responsibilities of caregiving and studying, and recurring conflicts between academic schedules and unpredictable care needs. Caregiving may lead to missed lectures, late submissions, difficulty completing placements, restricted course and university choices, and interruptions or reductions in study intensity (Knopf et al., 2022; Runacres et al., 2024; Day, 2021; Rawlinson, 2026). Quantitative studies have examined the effects of caregiving on academic performance. Norwegian young adult student carers were more likely than non-carers to report delayed study progress and repeated examination failure (Haugland et al., 2022), while a US study found that particular patterns of caring for ill or elderly people were associated with lower institutionally recorded GPA (Armstrong-Carter et al., 2022).

### 1.1 Demographics

Regarding the potential role of demographics as mental health risk factors, age and gender are the most commonly reported characteristics in the university student carer literature. Caregiving was found to be more common among female Norwegian and French young adult university students (Haugland et al., 2020; Chevrier et al., 2023). In contrast, a German national survey found no adjusted difference between women and men in the likelihood of being a student carer (Pirig et al., 2026). Regarding age, studies have tended to focus on one age group such as mature-aged student carers or young adult carers (Knopf et al., 2022; Runacres et al., 2024). In such cases both age groups have been identified as being at increased risk of adverse outcomes in the context of caregiving. The German national survey included students across a wider age range, and it found older students were more likely to provide care (Pirig et al., 2026). Studies have not examined the role of demographics as potential risk factors for mental health problems, addictive behaviors and academic performance in university student carers. Both reviews in the field have called for greater investigation of demographics in university student carers (Knopf et al., 2022; Runacres et al., 2024).

### 1.2 Education factors

Relations between educational variables such as degree programme (e.g., bachelor's, master's, doctoral), residential status (on-site, commuter, non-resident), and enrolment status (full-time, part-time, working student) and mental health and addictive behaviors in university student carers have not been examined. Variations in the demands of different degree programs are likely to differentially impact caregiving responsibilities and have roll-on effects on carer stress and mental health, addictive behaviors and academic performance (Knopf et al., 2022; Runacres et al., 2024). Similarly, caregiving is likely to intersect with choices about location of residence in relation to university attendance and decisions about capacity to take on full-time or part-time study and paid employment (Knopf et al., 2022; Runacres et al., 2024).

### 1.3 Caregiving context factors

Two important caregiving context factors that may influence mental health, addictive behaviors and academic performance in university student carers are the nature of the relationship between the carer and care recipient and the care recipient's type of health condition. Regarding the carer-care recipient relationship, most studies do not differentiate by relationship to the care recipient and have not examined associations with student carer mental health, addictive behaviors and academic performance. With respect to the care recipient's type of health condition, evidence suggests university student carers who provide care for someone with mental health problems report higher caregiving burden (Crandall et al., 2014) and poorer mental health (Haugland et al., 2023; van der Werf et al., 2019) than those caring for a person with physical illness. This finding is consistent with the robust evidence in the young adult carer and adult carer research showing carers of a person with mental illness report poorer mental health than those caring for someone with a physical illness (Haugland et al., 2023; Bremmers et al., 2025; Pakenham and Cox, 2014).

### 1.4 Caregiving responsibilities

No published study has directly examined associations between university student self-reported caregiving responsibilities and their mental health, addictive behaviors and academic performance. However, one study found that poorer mental health was associated with a higher number of hours spent on care responsibilities in a sample of Norwegian young adult university student carers (Haugland et al., 2020). This finding is consistent with strong evidence indicating an association between higher caregiving responsibilities and poorer mental health in young adult (Landi et al., 2022c; Fleitas Alfonzo et al., 2022) and adult carers (Kayaalp et al., 2021).

### 1.5 The present study

Despite student mental health problems emerging as a public health priority in higher education and preliminary evidence indicating that caregiving is a mental health risk factor in university students, there remains a dearth of research examining this issue. Many prior studies on university student carers have limited sampling to a particular age group (e.g., young adults), and or to the presence of an elderly family member in need of care or included as carers students parenting normally developing “healthy” children. These sampling limitations prevent the generalizability of findings to university student carers across the adult age spectrum who provide care to a family member with a serious health condition. In the present study we included students of all ages above 18 years and only include as carers students who identify a family household member who has a serious health condition. In addition, some prior studies have limited sampling to students who identify as carers. However, some carers, particularly young adult carers, do not self-identify as carers and are reluctant to disclose their caregiving experiences because of associated stigma, particularly in a higher education environment (Runacres et al., 2024). Investigating only those who self-identify as carer may exclude students who have substantial care responsibilities but do not identify as a carer. Hence, in the present study, we recruited university students with no requirement for participants to self-identify as a carer. Instead, students were identified as carer if they self-reported the presence of a family household member with a serious health condition.

The purpose of the present study is to investigate the mental health of student carers relative to non-carer peers and examine demographic, educational and caregiving variables as potential risk factors for university student carer mental health (anxiety, depression), addictive behaviors and academic performance problems. For the purposes of this study the mental health, addictive behavior and academic performance variables are from here on collectively referred to as outcomes where appropriate. This study was guided by two broad aims. The first aim was to examine differences in mental health, addictive behaviors and academic performance between university student carers and non-carers. We hypothesized that university student carers would report poorer mental health, more addictive behaviors and poorer academic performance than non-carers.

The second aim was to identify demographic, educational and caregiving variables associated with variations in university student carer mental health, addictive behaviors and academic performance. A limitation of relevant prior research is that studies have only examined relations between one or two of these potential mental health risk factors and student carer outcomes. Additionally, where more than one potential mental health risk factor has been considered, studies have failed to investigate the simultaneous or additive effects of these variables on student carer outcomes. Hence, in the present study we examined the incremental and simultaneous effects of these risk factors on carer mental health, addictive behaviors and academic performance. Guided by the distinction between relatively distal and stable contextual characteristics and more proximal changeable caregiving demands, predictors were examined hierarchically. Demographic variables were entered first, followed by educational variables. Relatively stable situational caregiving-context variables were then examined to determine whether they accounted for additional variability in carer outcomes beyond demographic and educational variables. Finally, caregiving responsibilities were examined as a more proximal and potentially modifiable predictor to determine whether they explained further variability in carer outcomes beyond the preceding factors.

Given the limited published data regarding associations between both demographic variables (sex, age, and nationality) and educational variables (degree programme, residential status, and enrolment status) and university student carer mental health, addictive behaviors and academic performance, examination of these associations was exploratory. Caregiving factors included caregiving context variables, namely the carer-care recipient relationship and care recipient's type of health condition and the more proximal and potentially malleable caregiving responsibilities. Due to the lack of published data on associations between the carer-care recipient relationship and carer mental health, addictive behaviors and academic performance, these relations were examined exploratorily. Previous research provided a stronger basis for predictions concerning relations between both the care recipient's type of health condition and caregiving responsibilities and carer outcomes. We hypothesized that university student carers living with family members affected by mental illness or substance-related problems would report poorer mental health, more addictive behaviors and poorer academic performance than those living with family members with physical illness. Finally, we expected that greater caregiving responsibilities would be associated with poorer mental health, more addictive behaviors and poorer academic performance.

## 2 Materials and methods

### 2.1 Participants and procedure

Participants were recruited between March and October 2025 via social media and the University of Bologna website, and the survey was administered through Qualtrics. Eligibility required current enrolment in a degree programme at the University of Bologna (Italy) and fluency in Italian. After the removal of 52 duplicate entries, the final sample comprised 2,533 university students. Participation was voluntary and no compensation was provided. All participants provided informed consent electronically before accessing the survey, after reading an information sheet. The study was designed and conducted in accordance with the World Medical Association Declaration of Helsinki and its subsequent revisions (World Medical Association, 2013) and was approved by the Ethics Committee of the University of Padua (Prot. n. 930-d, 17 March 2025).

### 2.2 Measures

#### 2.2.1 Demographic and educational variables

Participants reported their sex at birth (female, male, prefer not to say), age (18–22, 23–25, 26–35, >35 years), nationality, degree programme (bachelor's, master's, single-cycle, doctoral), residential status (on-site, commuter, non-resident), and enrolment status (full-time, part-time, working student).

#### 2.2.2 Caregiving context variables

Three caregiving-context variables were assessed: carer status, carer-care recipient relationship, and care recipient's type of health condition. Participants first indicated whether they currently lived with a parent with a serious health condition and were then asked whether they lived with any other family members with serious health conditions and could select all applicable relatives. University student carers were operationally defined as participants who reported currently living with at least one family member with a serious health condition; all remaining participants were classified as non-carers. The carer-care recipient relationship was ascertained from the nature of the carer's relationship to the family member or family members identified and included parent, sibling, grandparent, partner, and child. Participants also indicated all applicable health conditions affecting the family member or the family members in their household, including physical illness, physical disability, intellectual disability, mental health conditions, and alcohol or drug (substance-related) problems. These responses were used to derive the caregiving context variable care recipient's type of health condition. Multiple responses were permitted for both the carer-care recipient relationship and the care recipient's type of health condition because participants could report more than one affected family member and more than one type of health condition.

#### 2.2.3 Caregiving responsibilities

The level of caregiving responsibility was assessed with the validated Italian version of the Caregiving Responsibilities subscale of the Young Carer of Parents Inventory-Revised (YCOPI-R; Cox and Pakenham, 2014; Landi et al., 2022a). The subscale assesses the extent to which young people are expected to provide practical, emotional, and organizational support within the family related to the ill family member. It includes seven items rated on a five-point scale (0 = strongly disagree to 4 = strongly agree). Higher scores indicate greater caregiving responsibilities. Internal consistency in the present sample was good (Cronbach's α = 0.83).

#### 2.2.4 Anxiety symptoms

Anxiety symptoms were assessed with the validated Italian version of the Generalized Anxiety Disorder Scale (GAD-7; Spitzer et al., 2006; Bolgeo et al., 2023). Items are rated on a four-point scale (0 = not at all to 3 = nearly every day), yielding a total score from 0 to 21, with higher scores indicating greater anxiety severity. In the present sample, internal consistency was good (Cronbach's α = 0.89).

#### 2.2.5 Depressive symptoms

Depressive symptoms were assessed with the validated Italian version of the Patient Health Questionnaire (PHQ-9; Kroenke et al., 2001; Mazzotti et al., 2003). Items are rated on a four-point scale (0 = not at all to 3 = nearly every day), yielding a total score from 0 to 27, with higher scores indicating greater depression severity. In the present sample internal consistency was good (Cronbach's α = 0.87).

#### 2.2.6 Academic performance

Academic performance was assessed using an Italian adaptation of the 7-item scale originally developed by (Viskovich and Pakenham, 2020) for evaluating university student self-assessed academic performance. Participants rated their overall academic performance across multiple dimensions over the preceding 4 weeks using a ten-point Likert scale (1 = poor; 10 = excellent). A “not applicable” response option was available and was treated as missing. Higher scores indicated better academic performance. The academic performance scale has demonstrated excellent internal reliability, factorial and construct validity and sensitivity to intervention effects (Viskovich and Pakenham, 2020, 2018). In the present sample, the scale demonstrated good internal consistency (Cronbach's α = 0.90).

#### 2.2.7 Substance-related and behavioral addictions

Functional problems related to substance-related and behavioral addictions were assessed with the Screener for Substance and Behavioral Addictions (SSBA; Hodgins et al., 2023; Schluter et al., 2018, 2020). The SSBA applies to each target behavior four items, each capturing a core feature of problematic involvement—namely, excessive use, loss of control, use as a coping or functional strategy, and continued engagement despite negative consequences over the past 12 months. Items are rated on a five-point scale (0 = none of the time to 4 = all of the time), with an additional “prefer not to say” option coded as missing; the four item scores are summed to yield a domain score ranging from 0 to 16, with higher scores indicating greater problematic involvement. The SSBA was administered across 13 substance-related and behavioral domains: alcohol (Cronbach's α = 0.86), tobacco (Cronbach's α = 0.94), cannabis (Cronbach's α = 0.90), psychostimulants (Cronbach's α = 0.91), opioids (Cronbach's α = 0.87), shopping (Cronbach's α = 0.83), gambling (Cronbach's α = 0.79), video-gaming (Cronbach's α = 0.87), food (Cronbach's α = 0.91), sexual activity (Cronbach's α = 0.89), studying (Cronbach's α = 0.82), social-network use (Cronbach's α = 0.85), and physical exercise (Cronbach's α = 0.81). Domain retention and the operationalization of problematic involvement using cut-off scores are described in the Data Analysis section.

### 2.3 Data analysis

Analyses were conducted in SPSS 25 (IBM Corp, 2017), with R 4.3.1 (R Core Team, 2023) used to compute false discovery rate (FDR)-adjusted *q*-values. All tests were two-tailed (*p* < 0.05). Missing data were handled using listwise deletion within each model. Consequently, participants who preferred not to report their sex at birth (*n* = 18) and one participant with missing nationality data were excluded from models containing these variables.

Prior to the analyses, physical illness and physical disability were combined as *physical illness or disability*, and mental health conditions and intellectual disability were combined as *mental health condition or intellectual disability*. Because few participants reported an ill partner (*n* = 54) or child (*n* = 4), these care-recipients were combined as *other family member*. Caregiving context characteristics were represented by non-mutually exclusive binary indicators of the carer-care recipient relationship (parent, sibling, grandparent, or other family member) and the care recipient's type of health condition (physical illness/disability, mental health condition/intellectual disability, or substance-related problems). Accordingly, each coefficient represented the association of the presence vs. absence of that caregiving context characteristic, adjusted for the remaining indicators, rather than a comparison with a common reference group.

Thirteen study outcomes were examined: three continuous outcomes—depressive symptoms, anxiety symptoms, and academic performance—and 10 binary outcomes concerning problematic involvement in alcohol, tobacco, cannabis, shopping, videogaming, food consumption, sexual activity, studying, social-network use, and physical exercise.

After listwise deletion of participants with “not applicable” responses, academic-performance models included 2,092 participants in the total-sample analysis and 651 in the student-carer analysis. Psychostimulant and opioid involvement were excluded because validated cut-offs for problematic involvement were not available for these domains (Hodgins et al., 2023), and gambling was excluded because of its very low prevalence in the present sample. For the remaining SSBA domains, problematic involvement was operationalized using the cut-offs proposed by (Hodgins et al. 2023): tobacco ≥1; cannabis, videogaming, and sexual activity ≥3; alcohol, shopping, and physical exercise ≥4; and food consumption and studying ≥6. Because no validated cut-off was available for social-network use, this outcome was retained using an exploratory threshold of ≥6, in line with the threshold applied to other high-prevalence behavioral domains (e.g., food consumption, studying). “Prefer not to say” responses were treated as missing; therefore, analytic sample sizes varied slightly across binary outcomes and are reported in the corresponding tables.

As a preliminary validity check of the operational carer vs. non-carer classification, Caregiving Responsibilities scores were compared between carers and non-carers using Welch's *t*-test, with Cohen's *d* as the effect-size measure.

To address the first aim, separate regression models were estimated for each of the 13 study outcomes in the total sample. Each model included carer status as the main predictor, with non-carers as the reference group.

The second study aim was examined in the student-carer subsample using separate hierarchical regression analyses for each of the 13 study outcomes. Linear regressions were used for continuous outcomes and binary logistic regressions for dichotomous outcomes. Predictor blocks were entered into a hierarchically ordered sequence, moving from broad and relatively stable student demographic characteristics to increasingly specific and proximal features of the educational and caregiving contexts.

At Step 1, the three demographic variables—sex, age, and nationality—were entered simultaneously. Sex was coded as 0 = male and 1 = female; age was represented by three dummy variables for 23–25 years, 26–35 years, and >35 years, with 18–22 years as the reference category; and nationality was coded as 0 = non-Italian and 1 = Italian.

At Step 2, educational variables were added, comprising degree programme, residential status, and enrolment status. Degree programme was dummy-coded with bachelor's degree as the reference category and dummy indicators for master's degree, single-cycle degree, and PhD; residential status was dummy-coded with on-site students as the reference category and dummy indicators for commuter and non-resident students; and enrolment status was dummy-coded with full-time students as the reference category and dummy indicators for part-time student and working student.

At Step 3, caregiving-context variables were entered. These comprised the four non-mutually-exclusive care recipient family-status indicators—i.e., parent, sibling, grandparent, and other family member (i.e., partner and children)—and three non-mutually-exclusive care-recipient illness health-condition indicators: physical illness/disability, mental health condition/intellectual disability, and substance-related problems.

At Step 4, caregiving responsibilities were added as a continuous predictor representing a more proximal and potentially modifiable aspect of the caregiving role.

Before model estimation, linear regression models were examined for residual normality, homoscedasticity, influential observations, and multicollinearity. Logistic regression models were assessed for convergence, sparse cells, influential observations, and multicollinearity. Complete separation in the student-carer cannabis model, due to no cases among carers aged >35 years, was resolved by combining the two upper age groups (≥26 years); all other models retained the original age categories. Bivariate correlations were also computed in the carer subsample to describe zero-order associations among study variables and aid interpretation of hierarchical models. Correlations between predictors and outcomes are reported in Supplementary Tables S1, S2; intercorrelations among predictors are reported in Supplementary Table S3.

FDR adjusted *q*-values were computed using the Benjamini–Hochberg procedure (Benjamini and Hochberg, 1995). This procedure was selected because the analyses involved 13 outcomes and predefined predictor domains. While family-wise error-rate procedures are designed to control the probability of at least one Type I error within a family of tests, FDR correction controls the expected proportion of falsely rejected hypotheses among rejected hypotheses. Because FDR correction offers potential gains in power relative to family-wise error-rate control in multiple-testing contexts (Benjamini and Hochberg, 1995), it provided a suitable balance between multiplicity control and statistical power in the present analyses. For Aim 1, FDR correction was applied across the 13 tests of the association between carer status and each study outcome. For Aim 2, FDR correction was applied at two levels. At the primary level, FDR correction was applied separately to the block-change tests for each of the four predictor blocks across the 13 outcomes. This allowed to evaluate which domains—demographic, educational-context, caregiving-context, and caregiving responsibilities—showed an incremental contribution within the pre-specified hierarchical ordering. Exact *p* values for these block-change tests were obtained from the *F*-change statistics for linear regression models and from likelihood-ratio chi-square statistics for binary logistic regression models. At the secondary level, FDR correction was applied to final-model coefficients within four pre-specified coefficient families: demographic predictors, educational-context predictors, and two families nested within the broader caregiving-context block, namely indicators of the carer-care recipient relationship and indicators of the care recipient's type of illness or health condition. Final-model coefficients were used because they estimate the unique association of each predictor with the outcome after accounting for all other predictor domains. Caregiving responsibilities were corrected only at the block level because it was added at Step 4 as a single predictor, meaning that the block-change test and the coefficient tested the same substantive effect. Nominally significant associations that did not survive FDR correction were retained in the tables but interpreted cautiously.

## 3 Results

Sample characteristics are presented in Table 1. Most participants (70.5%) were female (male 28.8%; 0.7% preferred not to report their sex at birth) and Italian (94.0%). Most participants (94.9%) were 35 years or younger (18–22, 39.7%; 23–25, 32.7%; 26–35, 22.5%; >35 years 5.1). Almost one third of participants (30.1%, *n* = 762) reported living with a family member with a serious health condition. Among student carers, 46.5% reported living with a parent with a serious health condition only, 36.5% with another family member only, and 16.9% with both a parent and another family member with a serious health condition. Regarding the care recipient's type of health condition 56.2% of student carers reported living with a family member with a physical illness or disability, 65.2% with a mental health condition or intellectual disability, and 7.6% with alcohol or drug (substance-related) problems.

**Table 1:** Sample characteristics (*N* = 2,533).

Variable	*n*	%
Sex at birth		
Female	1,786	70.5
Male	729	28.8
Prefer not to report	18	0.7
Age band (years)		
18–22	1,005	39.7
23–25	828	32.7
26–35	570	22.5
>35	130	5.1
Nationality^a^		
Italian	2,380	94.0
Non-Italian	152	6.0
Degree programme		
Bachelor's	1,043	41.2
Master's	849	33.5
Single-cycle	366	14.4
Doctoral	275	10.9
Residential status		
On-site	504	19.9
Commuter	661	26.1
Non-resident	1,368	54.0
Enrolment status		
Full-time	1,816	71.7
Working student	598	23.6
Part-time	119	4.7
Presence of a family member with a health condition in the household		
No	1,771	69.9
Yes	762	30.1
Carer-care recipient relationship (among carers, *n* = 762)^b^		
Parent	483	63.4
Sibling	164	21.5
Grandparent	206	27.0
Other family member (i.e., partner and children)	58	7.6
Care recipient's type of illness (among carers, *n* = 762)^b^		
Physical illness or disability	428	56.2
Mental health condition or intellectual disability	497	65.2
Substance-related problems	58	7.6

a One participant had missing data on nationality.

b Percentages computed on the carer subsample (*n* = 762); categories are not mutually exclusive, as participants could report more than one family member and/or type of condition; percentages therefore do not sum to 100.

Descriptive statistics for all outcomes for the total sample and the carer and non-carer groups are presented in Table 2. In the total sample, the mean depressive symptom score was in the moderate range, whereas the mean anxiety symptom score was in the mild range. The prevalence of problematic involvement across SSBA domains varied widely, ranging from 7.7% for cannabis use to 54.6% for social network use. More than one quarter of participants scored above the threshold for tobacco use, studying, food consumption and physical exercise. Assumption checks revealed no violations; no influential outliers were identified.

**Table 2:** Descriptive statistics for caregiving responsibilities and outcomes in the total sample and by carer status.

Variable	*N*	Total sample *M*(*SD*)	Non-carers *M* (*SD*)	Carers *M* (*SD*)
Caregiving responsibilities (YCOPI-R)	2,533	1.68 (0.90)	1.55 (0.86)	1.98 (0.90)
Continuous outcomes				
Depressive symptoms (PHQ-9)	2,533	10.68 (5.98)	10.13 (5.81)	11.96 (6.17)
Anxiety symptoms (GAD-7)	2,533	9.71 (5.37)	9.31 (5.32)	10.63 (5.36)
Academic performance	2,092	5.19 (2.00)	5.26 (2.01)	5.05 (1.96)
Binary outcomes (SSBA)				
		***n*** **(%)**	***n*** **(%)**	***n*** **(%)**
Alcohol use	2,520	391 (15.5)	272 (15.4)	119 (15.7)
Tobacco use	2,519	829 (32.9)	569 (32.3)	260 (34.3)
Cannabis use	2,524	195 (7.7)	115 (6.5)	80 (10.5)
Shopping	2,523	321 (12.7)	204 (11.6)	117 (15.4)
Videogaming	2,526	562 (22.2)	369 (20.9)	193 (25.4)
Food consumption	2,518	677 (26.9)	422 (24.0)	255 (33.7)
Sexual activity	2,497	588 (23.5)	397 (22.7)	191 (25.6)
Studying	2,489	885 (35.6)	607 (34.8)	278 (37.3)
Social network use	2,518	1,376 (54.6)	932 (52.9)	444 (58.7)
Physical exercise	2,504	698 (27.9)	475 (27.1)	223 (29.7)

Total sample *N* = 2,533 (non-carers *n* = 1,771; carers *n* = 762). M, mean; SD, standard deviation; PHQ-9, patient health questionnaire-9; GAD-7, generalized anxiety disorder-7; YCOPI-R, young carers of parents inventory–revised; SSBA, screener for substance and behavioral addictions. Caregiving responsibilities are reported separately because they were used as a preliminary validity check of the operational carer vs. non-carer classification and were not treated as a study outcome. For continuous outcomes, valid *n* = 2,533 for PHQ-9, GAD-7, and YCOPI-R scores, and *n* = 2,092 for academic performance (non-carers *n* = 1,436; carers *n* = 656) due to “not applicable” responses on academic performance scale items. For SSBA domains, percentages are valid percentages computed on non-missing responses; valid n ranges from 2,489 to 2,526 in the total sample owing to “prefer not to say” responses coded as missing. Problematic involvement in each SSBA domain was defined as scoring at or above the cut-point.

### 3.1 Aim 1—differences in outcomes between university student carers and non-carers

Supporting the validity of the carer vs. non-carer grouping, university student carers reported significantly higher caregiving responsibilities (*M* = 1.98, *SD* = 0.90, *n* = 762) than non-carers (*M* = 1.55, *SD* = 0.86, *n* = 1,771), Welch's *t*_(1378.54)_ = 11.10, *p* < 0.001, mean difference = 0.43, 95% CI [0.35, 0.50], Cohen's *d* = 0.49.

Compared with non-carers, university student carers reported significantly higher depressive symptoms (*B* = 1.83, *SE* = 0.26, β = 0.14, 95% CI [1.33, 2.33], *q* < 0.001) and anxiety symptoms (*B* = 1.32, *SE* = 0.23, β = 0.11, 95% CI [0.87, 1.77], *q* < 0.001), and lower academic performance (*B* = −0.21, *SE* = 0.09, β = −0.05, 95% CI [−0.39, −0.02], *q* = 0.048). Regarding addictive behaviors, carer status was significantly associated with higher odds of problematic involvement in cannabis use (OR = 1.69, 95% CI [1.25, 2.28], *q* = 0.002), shopping (OR = 1.39, 95% CI [1.09, 1.78], *q* = 0.018), videogaming (OR = 1.29, 95% CI [1.06, 1.57], *q* = 0.024), food consumption (OR = 1.62, 95% CI [1.34, 1.95], *q* < 0.001), and social network use (OR = 1.26, 95% CI [1.06, 1.50], *q* = 0.018). No significant associations emerged for alcohol, tobacco, sexual activity, studying, or physical exercise (see Table 3).

**Table 3:** Differences in outcomes between university student carers and non-carers.

Outcome	*n*	*B*(*SE*)	β	95% CI	*p*	*q*
Continuous outcomes						
Depressive symptoms (PHQ-9)	2,533	1.83(0.26)	0.14	[1.33, 2.33]	< 0.001	< 0.001
Anxiety symptoms (GAD-7)	2,533	1.32(0.23)	0.11	[0.87, 1.77]	< 0.001	< 0.001
Academic performance	2,092	−0.21(0.09)	−0.05	[−0.39, −0.02]	0.030	0.048
**Binary outcomes (SSBA)**	***n***	**OR**		**95% CI**	***p***	
Alcohol use	2,520	1.02		[0.81, 1.29]	0.854	0.854
Tobacco use	2,519	1.10		[0.92, 1.31]	0.315	0.341
Cannabis use	2,524	1.69		[1.25, 2.28]	0.001	0.002
Shopping	2,523	1.39		[1.09, 1.78]	0.008	0.018
Videogaming	2,526	1.29		[1.06, 1.57]	0.013	0.024
Food consumption	2,518	1.62		[1.34, 1.95]	< 0.001	< 0.001
Sexual activity	2,497	1.17		[0.96, 1.43]	0.120	0.174
Studying	2,489	1.12		[0.93, 1.33]	0.231	0.273
Social network use	2,518	1.26		[1.06, 1.50]	0.008	0.018
Physical exercise	2,504	1.13		[0.94, 1.37]	0.194	0.252

PHQ-9, patient health questionnaire-9; GAD-7, generalized anxiety disorder-7; SSBA, screener for substance and behavioral addictions estimates are from separate regression models comparing carers with non-carers, with non-carers as the reference group. Linear regression was used for continuous outcomes and binary logistic regression for dichotomous outcomes. B, unstandardized regression coefficient; SE, standard error; β, standardized regression coefficient; OR, odds ratio; CI, confidence interval; q, Benjamini–Hochberg false discovery rate-adjusted *p* value. Analytic sample sizes vary because “not applicable” responses on academic performance and “prefer not to say” responses on the SSBA were coded as missing. Statistical significance was set at two-tailed *p* < 0.05.

### 3.2 Aim 2—associations between demographic, educational, caregiving context variables and caregiving responsibilities and carer outcomes

The hierarchical linear regression models predicting depressive symptoms, anxiety symptoms, and academic performance among university student carers are presented in Table 4, whereas the models predicting problematic addictive behaviors are presented in Table 5. Within the carer subsample, the four predictor domains showed distinct patterns of incremental contribution across outcomes. After FDR correction of the block-change tests, demographic predictors contributed to anxiety symptoms, academic performance, videogaming, sexual activity, and social network use. Educational predictors contributed to depressive symptoms, academic performance, tobacco use, and cannabis use. Caregiving context indicators contributed to depressive symptoms, anxiety symptoms, alcohol use, and tobacco use. Finally, caregiving responsibilities contributed to depressive symptoms, anxiety symptoms, food consumption, and physical exercise. When all predictors were entered, the final linear models were significant for all three continuous outcomes—depressive symptoms, anxiety symptoms and academic performance—and explained 7.7% to 10.2% of their variance. For addictive behavior outcomes, the final logistic models were significant for seven of the 10 outcomes—namely problematic involvement in alcohol, tobacco, cannabis, videogaming, food consumption, sexual activity and social network, with Nagelkerke pseudo-*R*^2^ values ranging from 0.031 to 0.277.

**Table 4:** Four-step hierarchical linear regression models predicting depressive symptoms, anxiety symptoms, and academic performance among university student carers.

Predictor	Depressive symptoms (PHQ-9) (*n* = 755)				Anxiety symptoms (GAD-7) (*n* = 755)				Academic performance (*n* = 651)			
	*B*(*SE*)	*β*	95%CI	*p*	*B*(*SE*)	*β*	95%CI	*p*	*B*(*SE*)	*β*	95%CI	*p*
Model 1: demographic covariates												
Sex (female)	1.17 (0.53)	0.08	[0.14, 2.21]	0.026	1.83 (0.45)	0.15	[0.94, 2.72]	< 0.001	−0.07 (0.18)	−0.02	[−0.43, 0.29]	0.693
Age 23–25	−0.01 (0.52)	0.00	[−1.03, 1.00]	0.978	0.48 (0.45)	0.04	[−0.40, 1.36]	0.282	−0.06 (0.17)	−0.01	[−0.40, 0.28]	0.733
Age 26–35	0.74 (0.61)	0.05	[−0.46, 1.95]	0.226	0.66 (0.53)	0.05	[−0.38, 1.70]	0.212	−0.65 (0.21)	−0.13	[−1.06, −0.23]	0.002
Age > 35	−1.32 (1.25)	−0.04	[−3.77, 1.12]	0.289	−1.23 (1.08)	−0.04	[−3.35, 0.88]	0.253	−0.32 (0.47)	−0.03	[−1.24, 0.61]	0.504
Nationality (Italian)	−1.72 (1.03)	−0.06	[−3.74, 0.30]	0.096	−0.39 (0.89)	−0.02	[−2.14, 1.36]	0.661	0.87 (0.35)	0.10	[0.18, 1.55]	0.013
Block fit: Δ*R*^2^*, F* change, *p*	Δ*R*^2^ = 0.015, *F*_(5, 749)_ = 2.22, *p* = 0.051, *q* = 0.094				Δ*R*^2^ = 0.026, *F*_(5, 749)_ = 4.05, *p* = 0.001, *q* = 0.004				Δ*R*^2^ = 0.030, *F*_(5, 645)_ = 3.94, *p* = 0.002, *q* = 0.004			
*Model R* ^2^	0.015				0.026				0.030			
Model 2: + educational covariates												
Sex (female)	0.98 (0.53)	0.07	[−0.05, 2.02]	0.063	1.73 (0.46)	0.14	[0.82, 2.63]	< 0.001	−0.04 (0.18)	−0.01	[−0.40, 0.32]	0.833
Age 23–25	0.68 (0.61)	0.05	[−0.51, 1.88]	0.264	0.60 (0.53)	0.05	[−0.44, 1.64]	0.258	−0.27 (0.20)	−0.07	[−0.67, 0.13]	0.187
Age 26–35	1.71 (0.72)	0.11	[0.29, 3.13]	0.018	0.85 (0.63)	0.06	[−0.39, 2.08]	0.178	−0.80 (0.24)	−0.16	[−1.28, −0.32]	0.001
Age > 35	−1.22 (1.30)	−0.04	[−3.78, 1.33]	0.348	−1.42 (1.13)	−0.05	[−3.64, 0.81]	0.211	−0.19 (0.48)	−0.02	[−1.13, 0.76]	0.699
Nationality (Italian)	−1.94 (1.06)	−0.07	[−4.01, 0.13]	0.066	−0.24 (0.92)	−0.01	[−2.05, 1.56]	0.790	0.73 (0.35)	0.08	[0.03, 1.42]	0.040
Master's degree	−1.46 (0.61)	−0.11	[−2.66, −0.25]	0.018	−0.39 (0.53)	−0.03	[−1.44, 0.66]	0.469	0.50 (0.21)	0.12	[0.10, 0.91]	0.016
Single-cycle degree	0.63 (0.68)	0.04	[−0.70, 1.96]	0.351	0.35 (0.59)	0.02	[−0.80, 1.51]	0.550	−0.30 (0.22)	−0.06	[−0.74, 0.14]	0.183
PhD	−2.38 (1.02)	−0.10	[−4.39, −0.38]	0.020	−0.86 (0.89)	−0.04	[−2.60, 0.88]	0.333	0.27 (0.42)	0.03	[−0.56, 1.09]	0.525
Commuter	0.33 (0.72)	0.02	[−1.08, 1.74]	0.642	−0.69 (0.63)	−0.06	[−1.91, 0.54]	0.273	0.36 (0.24)	0.08	[−0.12, 0.84]	0.145
Non-resident	0.61 (0.67)	0.05	[−0.71, 1.93]	0.363	−0.28 (0.58)	−0.03	[−1.42, 0.87]	0.637	0.28 (0.23)	0.07	[−0.16, 0.73]	0.214
Part-time student	1.44 (1.02)	0.05	[−0.57, 3.45]	0.159	1.88 (0.89)	0.08	[0.13, 3.63]	0.035	−0.74 (0.34)	−0.09	[−1.39, −0.08]	0.029
Working student	0.73 (0.57)	0.05	[−0.40, 1.85]	0.207	0.61 (0.50)	0.05	[−0.37, 1.59]	0.225	−0.35 (0.20)	−0.07	[−0.74, 0.03]	0.073
*Block fit:* Δ*R*^2^*, F change, p*	Δ*R*^2^ = 0.023, *F*_(7, 742)_ = 2.50, *p* = 0.015, *q* = 0.049				Δ*R*^2^ = 0.011, *F*_(7, 742)_ = 1.22, *p* = 0.289, *q* = 0.470				Δ*R*^2^ = 0.030, *F*_(7, 638)_ = 2.95, *p* = 0.005, *q* = 0.035			
*Model R* ^2^	0.037				0.037				0.060			
Model 3: + caregiving context covariates												
Sex (female)	0.81 (0.52)	0.06	[−0.21, 1.83]	0.121	1.59 (0.45)	0.13	[0.69, 2.48]	0.001	0.00 (0.18)	0.00	[−0.36, 0.36]	0.998
Age 23–25	0.40 (0.60)	0.03	[−0.79, 1.58]	0.508	0.36 (0.53)	0.03	[−0.68, 1.39]	0.497	−0.22 (0.21)	−0.05	[−0.63, 0.18]	0.280
Age 26–35	1.39 (0.72)	0.09	[−0.02, 2.80]	0.054	0.52 (0.63)	0.04	[−0.71, 1.75]	0.406	−0.76 (0.25)	−0.15	[−1.25, −0.28]	0.002
Age > 35	−1.59 (1.38)	−0.05	[−4.30, 1.11]	0.248	−2.19 (1.20)	−0.08	[−4.55, 0.17]	0.069	−0.15 (0.51)	−0.01	[−1.16, 0.86]	0.770
Nationality (Italian)	−2.04 (1.04)	−0.07	[−4.09, 0.01]	0.052	−0.26 (0.91)	−0.01	[−2.05, 1.53]	0.778	0.71 (0.36)	0.08	[0.01, 1.41]	0.046
Master's degree	−1.22 (0.61)	−0.09	[−2.41, −0.03]	0.045	−0.21 (0.53)	−0.02	[−1.25, 0.83]	0.688	0.47 (0.21)	0.11	[0.06, 0.88]	0.025
Single-cycle degree	0.69 (0.67)	0.04	[−0.62, 2.01]	0.302	0.43 (0.58)	0.03	[−0.72, 1.58]	0.461	−0.32 (0.23)	−0.06	[−0.76, 0.12]	0.159
PhD	−1.95 (1.01)	−0.08	[−3.93, 0.03]	0.054	−0.49 (0.88)	−0.02	[−2.22, 1.24]	0.578	0.26 (0.42)	0.03	[−0.56, 1.09]	0.534
Commuter	0.43 (0.71)	0.03	[−0.96, 1.82]	0.543	−0.52 (0.62)	−0.04	[−1.73, 0.70]	0.401	0.37 (0.25)	0.09	[−0.12, 0.85]	0.137
Non-resident	0.61 (0.67)	0.05	[−0.69, 1.92]	0.358	−0.24 (0.58)	−0.02	[−1.38, 0.90]	0.678	0.26 (0.23)	0.07	[−0.18, 0.71]	0.250
Part-time student	1.39 (1.01)	0.05	[−0.59, 3.38]	0.169	1.81 (0.88)	0.07	[0.08, 3.55]	0.041	−0.78 (0.34)	−0.09	[−1.44, −0.12]	0.021
Working student	0.48 (0.57)	0.03	[−0.64, 1.59]	0.404	0.38 (0.50)	0.03	[−0.60, 1.36]	0.445	−0.33 (0.20)	−0.07	[−0.72, 0.06]	0.093
Mental health/intellectual disability	2.06 (0.57)	0.16	[0.93, 3.18]	< 0.001	1.63 (0.50)	0.15	[0.65, 2.62]	0.001	−0.29 (0.20)	−0.07	[−0.68, 0.10]	0.147
Physical illness or disability	0.49 (0.57)	0.04	[−0.63, 1.62]	0.391	0.15 (0.50)	0.01	[−0.83, 1.14]	0.763	−0.13 (0.20)	−0.03	[−0.52, 0.26]	0.507
Substance-related problems	1.23 (0.87)	0.05	[−0.48, 2.94]	0.159	1.39 (0.76)	0.07	[−0.10, 2.88]	0.068	0.22 (0.29)	0.03	[−0.35, 0.80]	0.451
Carer of a parent	0.50 (0.69)	0.04	[−0.85, 1.85]	0.468	0.24 (0.60)	0.02	[−0.94, 1.42]	0.685	−0.19 (0.24)	−0.05	[−0.66, 0.27]	0.415
Carer of a sibling	1.12 (0.64)	0.07	[−0.14, 2.38]	0.081	0.34 (0.56)	0.03	[−0.76, 1.44]	0.548	−0.19 (0.22)	−0.04	[−0.62, 0.24]	0.378
Carer of a grandparent	−0.99 (0.73)	−0.07	[−2.43, 0.44]	0.174	−0.73 (0.64)	−0.06	[−1.98, 0.52]	0.253	0.22 (0.25)	0.05	[−0.27, 0.71]	0.384
Carer of another family member	0.70 (1.07)	0.03	[−1.41, 2.80]	0.516	1.20 (0.93)	0.06	[−0.64, 3.03]	0.202	−0.12 (0.37)	−0.02	[−0.85, 0.61]	0.751
*Block fit:* Δ*R*^2^*, F change, p*	Δ*R*^2^ = 0.041, *F*_(7, 735)_ = 4.70, *p* < 0.001, *q* < 0.001				Δ*R*^2^ = 0.035, *F*_(7, 735)_ = 3.91, *p* < 0.001, *q* = 0.002				Δ*R*^2^ = 0.013, *F*_(7, 631)_ = 1.22, *p* = 0.287, *q* = 0.379			
*Model R* ^2^	0.079				0.072				0.073			
Model 4: + caregiving responsibilities												
Sex (female)	0.50 (0.52)	0.03	[−0.52, 1.52]	0.338	1.30 (0.45)	0.10	[0.42, 2.19]	0.004^q^	0.04 (0.18)	0.01	[−0.32, 0.40]	0.839
Age 23–25	0.23 (0.60)	0.02	[−0.94, 1.41]	0.697	0.21 (0.52)	0.02	[−0.82, 1.23]	0.690	−0.20 (0.21)	−0.05	[−0.61, 0.20]	0.327
Age 26–35	1.09 (0.71)	0.07	[−0.31, 2.49]	0.128	0.25 (0.62)	0.02	[−0.97, 1.47]	0.687	−0.71 (0.25)	−0.14	[−1.20, −0.23]	0.004^q^
Age > 35	−2.06 (1.37)	−0.06	[−4.75, 0.62]	0.131	−2.61 (1.19)	−0.09	[−4.95, −0.28]	0.029	−0.08 (0.51)	−0.01	[−1.09, 0.92]	0.870
Nationality (Italian)	−2.32 (1.03)	−0.08	[−4.35, −0.29]	0.025	−0.51 (0.90)	−0.02	[−2.28, 1.26]	0.569	0.76 (0.36)	0.09	[0.06, 1.46]	0.033
Master's degree	−1.05 (0.60)	−0.08	[−2.23, 0.13]	0.080	−0.06 (0.52)	−0.01	[−1.09, 0.97]	0.909	0.45 (0.21)	0.11	[0.04, 0.85]	0.033
Single-cycle degree	0.58 (0.66)	0.04	[−0.72, 1.88]	0.378	0.33 (0.58)	0.02	[−0.80, 1.47]	0.565	−0.31 (0.23)	−0.06	[−0.75, 0.13]	0.168
PhD	−1.76 (1.00)	−0.07	[−3.72, 0.20]	0.078	−0.32 (0.87)	−0.02	[−2.03, 1.38]	0.711	0.25 (0.42)	0.03	[−0.57, 1.08]	0.544
Commuter	0.35 (0.70)	0.03	[−1.03, 1.72]	0.619	−0.59 (0.61)	−0.05	[−1.79, 0.61]	0.331	0.38 (0.25)	0.09	[−0.10, 0.86]	0.123
Non-resident	0.70 (0.66)	0.06	[−0.59, 1.99]	0.288	−0.16 (0.57)	−0.02	[−1.29, 0.96]	0.775	0.26 (0.23)	0.07	[−0.19, 0.70]	0.260
Part-time student	1.26 (1.00)	0.05	[−0.71, 3.22]	0.210	1.69 (0.87)	0.07	[−0.03, 3.40]	0.053	−0.76 (0.34)	−0.09	[−1.42, −0.10]	0.024
Working student	0.22 (0.57)	0.01	[−0.89, 1.33]	0.700	0.15 (0.49)	0.01	[−0.82, 1.12]	0.764	−0.30 (0.20)	−0.06	[−0.69, 0.09]	0.129
Mental health/intellectual disability	1.66 (0.57)	0.13	[0.54, 2.79]	0.004^q^	1.28 (0.50)	0.11	[0.30, 2.26]	0.011	−0.24 (0.20)	−0.06	[−0.63, 0.16]	0.238
Physical illness or disability	0.31 (0.57)	0.03	[−0.81, 1.43]	0.588	−0.01 (0.50)	0.00	[−0.99, 0.96]	0.976	−0.10 (0.20)	−0.03	[−0.49, 0.29]	0.615
Substance-related problems	1.12 (0.86)	0.05	[−0.56, 2.81]	0.191	1.29 (0.75)	0.06	[−0.18, 2.77]	0.085	0.24 (0.29)	0.03	[−0.33, 0.82]	0.406
Carer of a parent	0.15 (0.68)	0.01	[−1.20, 1.49]	0.830	−0.07 (0.60)	−0.01	[−1.25, 1.10]	0.902	−0.13 (0.24)	−0.03	[−0.60, 0.33]	0.573
Carer of a sibling	1.03 (0.63)	0.07	[−0.21, 2.27]	0.105	0.25 (0.55)	0.02	[−0.83, 1.34]	0.647	−0.18 (0.22)	−0.04	[−0.61, 0.25]	0.418
Carer of a grandparent	−1.15 (0.72)	−0.08	[−2.57, 0.27]	0.112	−0.87 (0.63)	−0.07	[−2.11, 0.37]	0.168	0.24 (0.25)	0.05	[−0.25, 0.73]	0.341
Carer of another family member	0.79 (1.06)	0.03	[−1.29, 2.87]	0.456	1.28 (0.92)	0.06	[−0.53, 3.09]	0.166	−0.12 (0.37)	−0.02	[−0.85, 0.61]	0.751
Caregiving responsibilities	1.12 (0.25)	0.16	[0.62, 1.62]	< 0.001^q^	1.01 (0.22)	0.17	[0.57, 1.44]	< 0.001^q^	−0.16 (0.09)	−0.07	[−0.34, 0.01]	0.072
*Block fit:* Δ*R*^2^*, F change, p*	Δ*R*^2^ = 0.024, *F*_(1, 734)_ = 19.42, *p* < 0.001, *q* < 0.001				Δ*R*^2^ = 0.025, *F*_(1, 734)_ = 20.73, *p* < 0.001, *q* < 0.001				Δ*R*^2^ = 0.005, *F*_(1, 630)_ = 3.24, *p* = 0.072, *q* = 0.188			
*Model R* ^2^	0.102				0.097				0.077			
*Overall Step 4 model*	*F*_(20, 734)_ = 4.18, *p* < 0.001				*F*_(20, 734)_ = 3.96, *p* < 0.001				*F*_(20, 630)_ = 2.64, *p* < 0.001			

Values are unstandardized regression coefficients (B) with standard errors, standardized coefficients (β) and 95% confidence intervals for the carer subsample. Reference categories: male sex; age 18–22; non-Italian nationality; bachelor's degree; on-site residential status; full-time enrolment status; non-working student. ΔR^2^ and F change index the improvement in model fit associated with that step; R^2^ is reported for the cumulative model at each step; Overall Step 4 model refers to the full final model including all predictors. Benjamini–Hochberg false discovery rate correction was applied at two levels. At the primary level, it was applied separately to the block-change tests of each of the four blocks across the 13 outcomes; these q values appear beside p in the block-fit rows. At the secondary level, it was applied to the final-model (Step 4) coefficients and coefficients surviving it are marked with a superscript q. Because Step 4 adds a single predictor, its block-change test and the YCOPI-R coefficient express the same substantive hypothesis and were corrected once only. Nominally significant associations that do not carry the marker remain visible in the table but should not be read as robust. Exact *p* values were obtained from the test statistics rather than from rounded output.

**Table 5:** Four-step hierarchical binary logistic regression models predicting problematic addictive behaviors among university student carers.

Predictor	Alcohol (*n* = 750)	Tobacco (*n* = 750)	Cannabis (*n* = 752)	Shopping (*n* = 753)	Videogaming (*n* = 753)	Food consumption (*n* = 749)	Sexual activity (*n* = 740)	Studying (*n* = 738)	Social network (*n* = 750)	Physical exercise (*n* = 745)
Model 1: demographic covariates										
Sex (female)	0.94 [0.59, 1.48]	0.97 [0.68, 1.38]	0.80 [0.48, 1.36]	**1.70 [1.00, 2.87]**	**0.34 [0.24, 0.50]**	1.19 [0.83, 1.71]	**0.13 [0.09, 0.18]**	1.22 [0.85, 1.73]	1.36 [0.96, 1.92]	**0.67 [0.47, 0.97]**
Age 23–25	0.91 [0.58, 1.43]	0.81 [0.57, 1.15]	1.14 [0.68, 1.92]	0.78 [0.49, 1.24]	0.79 [0.54, 1.17]	0.93 [0.65, 1.32]	0.76 [0.50, 1.15]	0.97 [0.69, 1.37]	0.74 [0.53, 1.04]	0.90 [0.63, 1.29]
Age 26–35	1.01 [0.60, 1.71]	1.06 [0.71, 1.59]	—	0.94 [0.55, 1.60]	0.87 [0.55, 1.37]	1.06 [0.70, 1.60]	**0.52 [0.31, 0.88]**	0.95 [0.63, 1.44]	**0.55 [0.37, 0.81]**	**0.58 [0.37, 0.92]**
Age > 35	0.40 [0.09, 1.79]	0.67 [0.27, 1.65]	—	1.03 [0.36, 2.94]	**0.23 [0.06, 0.83]**	1.15 [0.50, 2.63]	0.83 [0.30, 2.31]	1.00 [0.42, 2.40]	**0.08 [0.03, 0.25]**	1.37 [0.60, 3.14]
Age ≥ 26^a^	—	—	0.81 [0.43, 1.54]	—	—	—	—	—	—	—
Nationality (Italian)	1.19 [0.45, 3.18]	1.82 [0.84, 3.95]	1.36 [0.40, 4.60]	0.74 [0.32, 1.70]	0.55 [0.26, 1.14]	1.26 [0.62, 2.58]	1.25 [0.51, 3.05]	1.29 [0.63, 2.65]	0.84 [0.41, 1.70]	0.89 [0.43, 1.85]
*Block fit: LR *^2^${\text{χ}}_{\left(5\right)}^{2b}$, *p*	2.37, *p* = 0.797, *q* = 0.888	5.51, *p* = 0.357, *q* = 0.515	2.27, *p* = 0.687, *q* = 0.888	6.23, *p* = 0.284, *q* = 0.462	39.51, *p* < 0.001, *q* < 0.001	1.71, *p* = 0.888, *q* = 0.888	124.73, *p* < 0.001, *q* < 0.001	1.79, *p* = 0.877, *q* = 0.888	37.34, *p* < 0.001, *q* < 0.001	11.46, *p* = 0.043, *q* = 0.093
*Model R* ^2^ *N*	0.005	0.010	0.006	0.014	0.075	0.003	0.228	0.003	0.065	0.022
Model 2: + educational covariates										
Sex (female)	0.91 [0.57, 1.45]	0.94 [0.66, 1.36]	0.79 [0.46, 1.35]	1.69 [1.00, 2.88]	**0.34 [0.24, 0.50]**	1.13 [0.78, 1.64]	**0.11 [0.08, 0.17]**	1.21 [0.85, 1.74]	1.35 [0.95, 1.92]	**0.66 [0.46, 0.96]**
Age 23–25	0.98 [0.57, 1.68]	0.94 [0.62, 1.43]	1.39 [0.73, 2.65]	0.87 [0.50, 1.51]	1.00 [0.63, 1.58]	1.02 [0.67, 1.55]	0.92 [0.56, 1.51]	0.90 [0.60, 1.36]	0.72 [0.48, 1.09]	0.77 [0.50, 1.18]
Age 26–35	1.15 [0.61, 2.17]	1.17 [0.72, 1.92]	—	1.14 [0.61, 2.14]	1.17 [0.68, 2.00]	1.29 [0.79, 2.11]	0.60 [0.33, 1.11]	0.93 [0.57, 1.52]	**0.52 [0.32, 0.84]**	**0.42 [0.24, 0.73]**
Age > 35	0.42 [0.09, 1.97]	0.60 [0.23, 1.57]	—	1.13 [0.37, 3.40]	0.30 [0.08, 1.09]	1.13 [0.47, 2.72]	0.73 [0.24, 2.20]	1.00 [0.40, 2.51]	**0.08 [0.03, 0.26]**	0.96 [0.40, 2.33]
Age ≥ 26^a^	—	—	0.86 [0.39, 1.87]	—	—	—	—	—	—	—
Nationality (Italian)	1.14 [0.41, 3.17]	1.74 [0.78, 3.89]	1.45 [0.41, 5.11]	0.87 [0.37, 2.07]	0.64 [0.30, 1.37]	1.15 [0.55, 2.43]	1.02 [0.41, 2.57]	1.34 [0.64, 2.80]	0.89 [0.43, 1.84]	0.90 [0.42, 1.93]
Master's degree	0.77 [0.45, 1.32]	**0.65 [0.43, 0.99]**	0.62 [0.33, 1.16]	0.77 [0.45, 1.33]	0.72 [0.45, 1.15]	0.83 [0.55, 1.26]	0.65 [0.40, 1.07]	1.16 [0.77, 1.76]	1.05 [0.69, 1.59]	1.25 [0.81, 1.95]
Single-cycle degree	0.57 [0.30, 1.09]	**0.60 [0.37, 0.97]**	**0.24 [0.09, 0.63]**	0.87 [0.48, 1.58]	0.99 [0.60, 1.64]	0.95 [0.60, 1.50]	0.58 [0.33, 1.05]	1.47 [0.94, 2.31]	1.13 [0.71, 1.79]	1.14 [0.70, 1.85]
PhD	0.55 [0.20, 1.49]	0.65 [0.32, 1.32]	0.91 [0.32, 2.57]	0.43 [0.15, 1.23]	0.56 [0.25, 1.25]	**0.42 [0.19, 0.91]**	0.52 [0.21, 1.26]	1.00 [0.49, 2.03]	1.27 [0.64, 2.53]	1.73 [0.83, 3.63]
Commuter	0.86 [0.43, 1.71]	0.87 [0.52, 1.44]	0.86 [0.39, 1.89]	0.56 [0.30, 1.05]	0.92 [0.54, 1.55]	1.29 [0.78, 2.14]	1.43 [0.78, 2.64]	0.87 [0.54, 1.41]	0.90 [0.55, 1.47]	0.91 [0.55, 1.52]
Non-resident	1.54 [0.83, 2.86]	1.46 [0.91, 2.32]	1.37 [0.67, 2.79]	0.79 [0.45, 1.39]	0.72 [0.44, 1.18]	1.32 [0.82, 2.12]	1.57 [0.89, 2.79]	0.85 [0.54, 1.33]	0.89 [0.56, 1.40]	0.86 [0.53, 1.38]
Part-time student	1.90 [0.85, 4.25]	1.52 [0.76, 3.04]	**3.08 [1.34, 7.04]**	1.65 [0.74, 3.67]	1.19 [0.56, 2.50]	1.61 [0.81, 3.19]	1.84 [0.84, 4.07]	0.72 [0.36, 1.47]	1.63 [0.77, 3.43]	1.36 [0.67, 2.75]
Working student	1.32 [0.80, 2.18]	**1.64 [1.12, 2.42]**	1.47 [0.82, 2.65]	1.13 [0.68, 1.89]	0.78 [0.49, 1.22]	1.37 [0.93, 2.02]	**1.80 [1.12, 2.88]**	0.89 [0.60, 1.32]	0.91 [0.62, 1.33]	1.49 [1.00, 2.24]
*Block fit: LR χ*^2^_(7)_, *p*	11.97, *p* = 0.101, *q* = 0.220	18.87, *p* = 0.009, *q* = 0.037	20.11, *p* = 0.005, *q* = 0.035	7.61, *p* = 0.368, *q* = 0.504	7.03, *p* = 0.426, *q* = 0.504	10.93, *p* = 0.142, *q* = 0.264	14.67, *p* = 0.041, *q* = 0.105	4.58, *p* = 0.711, *q* = 0.770	3.06, *p* = 0.880, *q* = 0.880	7.41, *p* = 0.387, *q* = 0.504
*Model R* ^2^ *N*	0.033	0.044	0.060	0.032	0.088	0.023	0.253	0.012	0.071	0.036
Model 3: + caregiving context covariates										
Sex (female)	0.87 [0.54, 1.40]	0.89 [0.62, 1.29]	0.73 [0.42, 1.26]	1.64 [0.96, 2.80]	**0.33 [0.23, 0.48]**	1.10 [0.76, 1.59]	**0.10 [0.07, 0.15]**	1.25 [0.87, 1.80]	1.31 [0.92, 1.86]	**0.67 [0.46, 0.97]**
Age 23–25	0.91 [0.52, 1.59]	0.85 [0.55, 1.31]	1.26 [0.65, 2.43]	0.82 [0.47, 1.44]	0.98 [0.62, 1.56]	0.96 [0.63, 1.47]	0.87 [0.52, 1.43]	0.96 [0.63, 1.45]	0.70 [0.46, 1.06]	0.79 [0.51, 1.21]
Age 26–35	1.02 [0.53, 1.95]	1.04 [0.62, 1.72]	—	1.06 [0.56, 2.01]	1.16 [0.67, 2.00]	1.20 [0.73, 1.98]	0.55 [0.29, 1.02]	1.00 [0.61, 1.64]	**0.51 [0.31, 0.82]**	**0.44 [0.25, 0.76]**
Age > 35	0.36 [0.07, 1.79]	0.59 [0.21, 1.64]	—	1.01 [0.31, 3.33]	0.28 [0.07, 1.10]	0.96 [0.37, 2.48]	0.54 [0.16, 1.80]	1.14 [0.42, 3.09]	**0.08 [0.02, 0.25]**	1.09 [0.41, 2.88]
Age ≥ 26^a^	—	—	0.76 [0.34, 1.68]	—	—	—	—	—	—	—
Nationality (Italian)	1.23 [0.44, 3.49]	1.91 [0.84, 4.32]	1.60 [0.45, 5.71]	0.86 [0.36, 2.06]	0.61 [0.28, 1.32]	1.14 [0.54, 2.41]	0.99 [0.39, 2.49]	1.21 [0.57, 2.58]	0.90 [0.43, 1.90]	0.84 [0.39, 1.83]
Master's degree	0.80 [0.47, 1.39]	0.71 [0.46, 1.08]	0.65 [0.34, 1.22]	0.82 [0.47, 1.43]	0.72 [0.45, 1.16]	0.87 [0.57, 1.33]	0.67 [0.40, 1.10]	1.10 [0.72, 1.68]	1.06 [0.70, 1.61]	1.24 [0.80, 1.94]
Single-cycle degree	0.58 [0.30, 1.13]	0.63 [0.39, 1.03]	**0.24 [0.09, 0.65]**	0.90 [0.49, 1.65]	0.98 [0.59, 1.64]	0.97 [0.61, 1.55]	0.58 [0.32, 1.05]	1.40 [0.89, 2.21]	1.13 [0.71, 1.80]	1.12 [0.69, 1.82]
PhD	0.62 [0.22, 1.71]	0.75 [0.36, 1.54]	1.08 [0.38, 3.09]	0.46 [0.16, 1.34]	0.57 [0.25, 1.29]	**0.45 [0.20, 0.99]**	0.55 [0.22, 1.38]	0.95 [0.46, 1.95]	1.35 [0.67, 2.71]	1.70 [0.81, 3.58]
Commuter	0.93 [0.46, 1.88]	0.92 [0.55, 1.54]	0.98 [0.44, 2.19]	0.60 [0.32, 1.13]	0.94 [0.55, 1.60]	1.39 [0.83, 2.31]	1.50 [0.81, 2.79]	0.86 [0.53, 1.39]	0.95 [0.58, 1.55]	0.89 [0.53, 1.48]
Non-resident	1.48 [0.79, 2.79]	1.50 [0.93, 2.41]	1.51 [0.73, 3.12]	0.84 [0.47, 1.49]	0.75 [0.46, 1.24]	1.40 [0.87, 2.28]	1.66 [0.93, 2.97]	0.81 [0.51, 1.27]	0.94 [0.59, 1.48]	0.82 [0.51, 1.32]
Part-time student	1.71 [0.75, 3.90]	1.48 [0.73, 3.03]	**3.13 [1.33, 7.36]**	1.71 [0.76, 3.83]	1.26 [0.59, 2.68]	1.66 [0.83, 3.33]	1.96 [0.87, 4.42]	0.69 [0.34, 1.42]	1.71 [0.80, 3.64]	1.34 [0.66, 2.74]
Working student	1.35 [0.80, 2.26]	**1.66 [1.12, 2.46]**	1.50 [0.82, 2.76]	1.07 [0.63, 1.80]	0.75 [0.47, 1.19]	1.29 [0.87, 1.91]	**1.72 [1.07, 2.77]**	0.88 [0.59, 1.32]	0.90 [0.61, 1.33]	1.50 [0.99, 2.26]
Mental health/intellectual disability	0.84 [0.48, 1.48]	1.07 [0.71, 1.62]	1.00 [0.53, 1.89]	1.41 [0.84, 2.36]	**1.63 [1.05, 2.53]**	**1.62 [1.08, 2.41]**	**2.24 [1.37, 3.68]**	1.12 [0.75, 1.66]	1.47 [0.99, 2.19]	1.02 [0.67, 1.56]
Physical illness or disability	**0.46 [0.26, 0.79]**	0.81 [0.54, 1.22]	0.62 [0.33, 1.17]	1.38 [0.83, 2.29]	1.50 [0.97, 2.33]	1.37 [0.92, 2.03]	1.45 [0.90, 2.34]	1.03 [0.69, 1.53]	1.09 [0.73, 1.63]	1.08 [0.71, 1.64]
Substance-related problems	1.93 [1.00, 3.75]	**2.35 [1.31, 4.24]**	1.87 [0.88, 3.98]	1.84 [0.92, 3.66]	1.19 [0.61, 2.33]	1.76 [0.98, 3.15]	1.25 [0.62, 2.54]	1.09 [0.60, 1.99]	1.18 [0.64, 2.17]	1.01 [0.53, 1.95]
Carer of a parent	0.89 [0.46, 1.69]	1.17 [0.72, 1.92]	1.37 [0.59, 3.18]	1.07 [0.56, 2.03]	1.02 [0.60, 1.76]	1.17 [0.72, 1.88]	1.10 [0.61, 1.99]	0.64 [0.40, 1.03]	1.03 [0.65, 1.64]	0.80 [0.49, 1.32]
Carer of a sibling	0.89 [0.49, 1.62]	0.93 [0.59, 1.47]	0.51 [0.24, 1.09]	0.95 [0.53, 1.69]	0.84 [0.51, 1.38]	1.02 [0.66, 1.60]	0.86 [0.50, 1.50]	0.91 [0.58, 1.44]	0.66 [0.43, 1.03]	1.24 [0.78, 1.97]
Carer of a grandparent	0.92 [0.46, 1.85]	0.74 [0.44, 1.26]	0.70 [0.29, 1.69]	0.88 [0.45, 1.73]	0.88 [0.49, 1.55]	0.89 [0.53, 1.49]	0.70 [0.37, 1.33]	1.13 [0.68, 1.88]	0.78 [0.48, 1.29]	0.98 [0.58, 1.67]
Carer of another family member	1.05 [0.40, 2.75]	0.89 [0.42, 1.92]	0.86 [0.27, 2.74]	1.28 [0.50, 3.26]	1.06 [0.46, 2.46]	1.48 [0.71, 3.06]	1.36 [0.55, 3.36]	0.71 [0.32, 1.56]	0.85 [0.41, 1.79]	0.79 [0.35, 1.75]
*Block fit: LR χ*^2^_(7)_, *p*	20.10, *p* = 0.005, *q* = 0.019	19.91, *p* = 0.006, *q* = 0.019	16.23, *p* = 0.023, *q* = 0.060	5.38, *p* = 0.614, *q* = 0.665	6.24, *p* = 0.512, *q* = 0.605	11.67, *p* = 0.112, *q* = 0.208	14.37, *p* = 0.045, *q* = 0.098	9.97, *p* = 0.191, *q* = 0.310	8.49, *p* = 0.291, *q* = 0.379	3.29, *p* = 0.857, *q* = 0.857
*Model R* ^2^ *N*	0.077	0.079	0.102	0.044	0.100	0.044	0.276	0.030	0.085	0.042
Sex (female)	0.86 [0.53, 1.39]	0.87 [0.60, 1.26]	0.72 [0.41, 1.25]	1.58 [0.92, 2.70]	**0.33**^**q**^ **[0.22, 0.48]**	1.02 [0.70, 1.49]	**0.10**^**q**^ **[0.07, 0.15]**	1.22 [0.84, 1.76]	1.32 [0.93, 1.89]	**0.62 [0.43, 0.90]**
Age 23–25	0.91 [0.52, 1.58]	0.83 [0.54, 1.28]	1.25 [0.65, 2.42]	0.80 [0.46, 1.41]	0.97 [0.61, 1.55]	0.92 [0.60, 1.41]	0.85 [0.52, 1.41]	0.94 [0.62, 1.43]	0.70 [0.46, 1.07]	0.75 [0.48, 1.16]
Age 26–35	1.01 [0.52, 1.94]	1.00 [0.60, 1.67]	—	1.02 [0.54, 1.94]	1.13 [0.66, 1.96]	1.12 [0.68, 1.85]	0.54 [0.29, 1.00]	0.97 [0.59, 1.60]	**0.51 [0.31, 0.84]**	**0.40**^**q**^ **[0.23, 0.70]**
Age > 35	0.35 [0.07, 1.76]	0.55 [0.20, 1.56]	—	0.95 [0.29, 3.17]	0.27 [0.07, 1.06]	0.86 [0.33, 2.23]	0.52 [0.15, 1.74]	1.10 [0.40, 2.99]	**0.08**^**q**^ **[0.02, 0.26]**	0.98 [0.37, 2.61]
Age ≥ 26 ^a^	—	—	0.75 [0.34, 1.67]	—	—	—	—	—	—	—
Nationality (Italian)	1.22 [0.43, 3.48]	1.87 [0.82, 4.26]	1.59 [0.44, 5.70]	0.84 [0.35, 2.02]	0.60 [0.27, 1.30]	1.07 [0.50, 2.29]	0.96 [0.38, 2.43]	1.19 [0.56, 2.52]	0.91 [0.44, 1.92]	0.77 [0.35, 1.68]
Master's degree	0.81 [0.47, 1.40]	0.72 [0.47, 1.10]	0.65 [0.35, 1.23]	0.84 [0.48, 1.47]	0.73 [0.46, 1.17]	0.91 [0.59, 1.39]	0.68 [0.41, 1.12]	1.12 [0.73, 1.70]	1.05 [0.69, 1.60]	1.31 [0.83, 2.05]
Single-cycle degree	0.58 [0.30, 1.13]	0.63 [0.39, 1.02]	**0.23 [0.09, 0.64]**	0.89 [0.49, 1.63]	0.97 [0.58, 1.62]	0.95 [0.59, 1.51]	0.57 [0.31, 1.04]	1.39 [0.88, 2.19]	1.13 [0.71, 1.81]	1.10 [0.67, 1.79]
PhD	0.63 [0.23, 1.73]	0.76 [0.37, 1.58]	1.09 [0.38, 3.12]	0.47 [0.16, 1.36]	0.58 [0.26, 1.31]	0.46 [0.21, 1.03]	0.56 [0.22, 1.40]	0.97 [0.47, 2.00]	1.34 [0.67, 2.68]	1.83 [0.87, 3.87]
Commuter	0.93 [0.46, 1.87]	0.91 [0.55, 1.53]	0.98 [0.44, 2.19]	0.59 [0.31, 1.12]	0.94 [0.55, 1.59]	1.38 [0.82, 2.31]	1.50 [0.81, 2.77]	0.85 [0.52, 1.38]	0.95 [0.58, 1.56]	0.87 [0.52, 1.45]
Non-resident	1.49 [0.79, 2.80]	1.52 [0.94, 2.44]	1.51 [0.73, 3.13]	0.85 [0.48, 1.52]	0.76 [0.46, 1.25]	1.45 [0.89, 2.36]	1.68 [0.94, 3.00]	0.81 [0.51, 1.27]	0.94 [0.59, 1.48]	0.83 [0.51, 1.35]
Model 4: + caregiving responsibilities										
Part-time student	1.70 [0.75, 3.88]	1.45 [0.71, 2.96]	**3.12 [1.33, 7.33]**	1.67 [0.74, 3.77]	1.24 [0.58, 2.65]	1.58 [0.78, 3.20]	1.95 [0.87, 4.39]	0.68 [0.33, 1.41]	1.72 [0.81, 3.67]	1.30 [0.64, 2.67]
Working student	1.33 [0.79, 2.24]	**1.61 [1.08, 2.41]**	1.49 [0.81, 2.74]	1.04 [0.61, 1.76]	0.74 [0.46, 1.17]	1.22 [0.82, 1.81]	**1.67 [1.03, 2.71]**	0.87 [0.58, 1.29]	0.91 [0.62, 1.35]	1.41 [0.93, 2.14]
Mental health/intellectual disability	0.83 [0.47, 1.46]	1.03 [0.68, 1.56]	0.99 [0.52, 1.88]	1.35 [0.80, 2.27]	**1.59 [1.02, 2.48]**	1.47 [0.98, 2.22]	**2.17**^**q**^ **[1.32, 3.58]**	1.08 [0.72, 1.62]	**1.50 [1.00, 2.24]**	0.93 [0.60, 1.42]
Physical illness or disability	**0.45**^**q**^ **[0.26, 0.78]**	0.79 [0.53, 1.19]	0.62 [0.33, 1.16]	1.35 [0.81, 2.25]	1.48 [0.95, 2.30]	1.31 [0.88, 1.96]	1.44 [0.89, 2.32]	1.02 [0.68, 1.52]	1.10 [0.74, 1.65]	1.03 [0.67, 1.57]
Substance-related problems	1.93 [0.99, 3.73]	**2.34**^**q**^ **[1.30, 4.21]**	1.86 [0.87, 3.97]	1.83 [0.92, 3.64]	1.18 [0.61, 2.31]	1.73 [0.96, 3.12]	1.24 [0.61, 2.52]	1.09 [0.60, 1.98]	1.18 [0.64, 2.18]	0.97 [0.50, 1.88]
Carer of a parent	0.87 [0.46, 1.67]	1.13 [0.69, 1.85]	1.35 [0.58, 3.15]	1.02 [0.53, 1.94]	1.00 [0.58, 1.73]	1.07 [0.66, 1.74]	1.07 [0.59, 1.94]	0.62 [0.38, 1.01]	1.04 [0.65, 1.66]	0.74 [0.45, 1.22]
Carer of a sibling	0.89 [0.49, 1.62]	0.92 [0.59, 1.45]	0.51 [0.24, 1.09]	0.93 [0.52, 1.66]	0.83 [0.50, 1.37]	1.01 [0.64, 1.58]	0.86 [0.49, 1.48]	0.91 [0.58, 1.43]	0.66 [0.43, 1.03]	1.22 [0.76, 1.94]
Carer of a grandparent	0.91 [0.45, 1.84]	0.73 [0.43, 1.24]	0.69 [0.29, 1.68]	0.86 [0.43, 1.69]	0.87 [0.49, 1.54]	0.86 [0.51, 1.44]	0.69 [0.36, 1.31]	1.12 [0.67, 1.87]	0.79 [0.48, 1.29]	0.96 [0.56, 1.64]
Carer of another family member	1.06 [0.40, 2.77]	0.90 [0.42, 1.93]	0.86 [0.27, 2.75]	1.29 [0.51, 3.29]	1.07 [0.46, 2.49]	1.53 [0.73, 3.18]	1.38 [0.56, 3.39]	0.71 [0.32, 1.56]	0.85 [0.41, 1.78]	0.80 [0.36, 1.79]
Caregiving responsibilities	1.04 [0.82, 1.33]	1.13 [0.94, 1.36]	1.04 [0.78, 1.38]	1.15 [0.91, 1.46]	1.08 [0.89, 1.32]	**1.32**^**q**^ **[1.10, 1.59]**	1.11 [0.89, 1.38]	1.09 [0.91, 1.30]	0.96 [0.80, 1.15]	**1.31**^**q**^ **[1.08, 1.59]**
*Block fit: LR χ*^2^_(1)_, *p*	0.13, *p* = 0.718, *q* = 0.778	1.60, *p* = 0.206, *q* = 0.446	0.07, *p* = 0.789, *q* = 0.788	1.37, *p* = 0.241, *q* = 0.448	0.62, *p* = 0.430, *q* = 0.559	8.89, *p* = 0.003, *q* = 0.012	0.80, *p* = 0.371, *q* = 0.536	0.88, *p* = 0.349, *q* = 0.536	0.20, *p* = 0.655, *q* = 0.774	7.74, *p* = 0.005, *q* = 0.018
*Model R* ^2^ *N*	0.077	0.082	0.102	0.047	0.101	0.060	0.277	0.031	0.085	0.056
*Overall Step 4 model*	LRχ^2^(20) = 34.57, *p* = 0.023	LRχ^2^(20) = 45.89, *p* = 0.001	LRχ^2^(19) = 38.68, *p* = 0.005	LRχ^2^(20) = 20.59, *p* = 0.422	LRχ^2^(20) = 53.40, *p* < 0.001	LRχ^2^(20) = 33.20, *p* = 0.032	LRχ^2^(20) = 154.56, *p* < 0.001	LRχ^2^(20) = 17.22, *p* = 0.639	LRχ^2^(20) = 49.09, *p* < 0.001	LRχ^2^(20) = 29.89, *p* = 0.072

Values are odds ratios (ORs) with 95% confidence intervals for the carer subsample; boldface indicates nominal statistical significance (two-tailed *p* < 0.05); equivalently, 95% confidence intervals not including 1 denote significant coefficients. Analytic sample sizes vary across outcomes owing to listwise deletion [“prefer not to say” responses on the Screener for Substance and Behavioral Addictions (SSBA) coded as missing; participants who preferred not to report their sex at birth or with missing data on nationality excluded]. Problematic involvement in each domain was defined as scoring at or above the clinical cut-point on the SSBA. Reference categories: male sex; age 18–22; non-Italian nationality; bachelor's degree; on-site residential status; full-time enrolment status; non-working student. Block LR χ^2^ indexes the improvement in model fit associated with that step; R^2^N is Nagelkerke pseudo-R^2^ for the cumulative model at each step. Overall Step 4 model refers to the full final logistic model including all predictors. Benjamini–Hochberg false discovery rate correction was applied at two levels. At the primary level, it was applied separately to the block-change tests of each of the four blocks across the 13 outcomes; these q values appear beside p in the block-fit rows. At the secondary level, it was applied to the final-model (Step 4) coefficients and coefficients surviving it are marked with a superscript q. Because Step 4 adds a single predictor, its block-change test and the Young Carers of Parents Inventory–Revised (YCOPI-R) coefficient express the same substantive hypothesis and were corrected once only. Nominally significant associations that do not carry the marker remain visible in the table but should not be read as robust.

a For the cannabis model, the two upper age bands were collapsed (≥26 years) owing to complete separation on the > 35 indicator in the original specification.

b df = 4 for the cannabis model.

Secondary FDR-corrected analyses of final-model coefficients clarified which specific predictors accounted for these patterns (all *q* < 0.05). Among demographic predictors, female sex was associated with higher anxiety symptoms (*B* = 1.30, *SE* = 0.45, β = 0.10, 95% CI [0.42, 2.19]) and lower odds of problematic videogaming (OR = 0.33, 95% CI [0.22, 0.48]) and sexual activity (OR = 0.10, 95% CI [0.07, 0.15]). Age 26–35 was associated with lower academic performance (*B* = −0.71, *SE* = 0.25, β = −0.14, 95% CI [−1.20, −0.23]) and lower odds of problematic physical exercise (OR = 0.40, 95% CI [0.23, 0.70]); and age above 35 was associated with lower odds of problematic social network use (OR = 0.08, 95% CI [0.02, 0.26]). No individual educational variable coefficient survived FDR correction, despite the significant incremental contribution of the educational block for selected outcomes. Among caregiving-context indicators, mental health condition or intellectual disability affecting the care recipient was associated with higher depressive symptoms (*B* = 1.66, *SE* = 0.57, β = 0.13, 95% CI [0.54, 2.79]) and higher odds of problematic sexual activity (OR = 2.17, 95% CI [1.32, 3.58]); physical illness or disability was associated with lower odds of problematic alcohol use (OR = 0.45, 95% CI [0.26, 0.78]); substance-related problems were associated with higher odds of problematic tobacco use (OR = 2.34, 95% CI [1.30, 4.21]). No carer-care recipient relationship indicator survived FDR correction. Greater caregiving responsibilities were associated with higher depressive symptoms (*B* = 1.12, *SE* = 0.25, β = 0.16, 95% CI [0.62, 1.62]), higher anxiety symptoms (*B* = 1.01, *SE* = 0.22, β = 0.17, 95% CI [0.57, 1.44]), and higher odds of problematic food consumption (OR = 1.32, 95% CI [1.10, 1.59]) and physical exercise (OR = 1.31, 95% CI [1.08, 1.59]).

## 4 Discussion

This cross-sectional study examined 2,533 university students, of whom 762 (30.1%) were classified as student carers because they lived with a family member with a serious health condition. Our purpose was to investigate the mental health of university student carers relative to non-carer peers and examine the role of demographic, educational and caregiving variables as potential risk factors for student carer mental health (anxiety, depression), addictive behaviors and academic performance problems. Regarding the first study aim, as predicted carers reported higher depressive and anxiety symptoms and lower academic performance than non-carers and were more likely to report problematic involvement in cannabis use, shopping, videogaming, food consumption, and social network use. With respect to the second study aim, each of the four predictor domains (demographics, educational, caregiving context and caregiving responsibilities) made a significant incremental contribution to at least one study outcome. When considered jointly, these domains were significantly associated with depressive symptoms, anxiety symptoms, academic performance, and seven of the 10 addictive-behavior outcomes. This finding highlights the importance of accounting for how demographic, educational-context, and caregiving variables are jointly associated with poorer outcomes in university student carers. Importantly, after adjusting for demographic, educational and caregiving context variables, greater caregiving responsibilities were associated with higher depressive and anxiety symptoms, as well as higher odds of problematic food consumption and physical exercise. This finding highlights the role of caregiving responsibilities as a key risk marker for poorer mental health among university student carers.

Student carers reported higher depressive and anxiety symptoms than non-carer peers, a finding consistent with previous studies on university student carers (Haugland et al., 2020; Greene et al., 2017). These results should also be interpreted against the broader context of psychological distress among Italian university students. Using the same screening measures, Riboldi et al. (2025) reported mean levels of depressive and anxiety symptoms broadly comparable to those observed in the present total sample. This suggests that student carers may represent a subgroup in which an already substantial mental health burden in the wider university population is further intensified by caregiving-related demands. However, this comparison is descriptive and should be interpreted cautiously.

Student carers also reported lower self-assessed academic performance than non-carer peers. Although the magnitude of this difference was small, this finding is consistent with previous evidence of academic difficulties among university student carers. In a large Norwegian study, young adult carers were more likely than non-carers to report delayed study progress and repeated examination failure (Haugland et al., 2022). Similarly, a large US study found that among students caring for an ill or elderly person, providing care on 3–5 days per week was associated with lower institutionally recorded GPA (Armstrong-Carter et al., 2022). Qualitative studies and reviews further suggest that caregiving can interfere with university study through conflicts between unpredictable care demands and academic schedules, reduced time for study, missed classes, and difficulties meeting assessment and placement requirements (Knopf et al., 2022; Runacres et al., 2024; Day, 2021; Rawlinson, 2026). These competing demands may help explain the lower academic performance observed in the present study. However, longitudinal research is needed to establish temporal ordering and to determine whether specific caregiving demands are associated with academic difficulties.

In line with Hodgins et al. (2023), who showed that problematic involvement among university students extends beyond substance use to several behavioral domains, carer status in our sample was associated with greater problematic involvement in cannabis use, shopping, videogaming, food consumption, and social network use. This is noteworthy given that only one study (Yiengprugsawan et al., 2012) has examined addictive behaviors among university student carers, reporting higher levels of problematic smoking and drinking and higher body mass index among Thai university student carers than among non-carer peers. In contrast, alcohol and tobacco did not differentiate carers from non-carers in our sample. This domain-specific pattern may suggest that student caregiving is less closely linked to substance use broadly than to readily accessible forms of emotion regulation and reinforcement. Many of the behaviors over-represented among carers can be engaged privately, often within the home environment, and with relatively limited demands on time, mobility, or social availability. They may therefore provide rapid reward, distraction, or emotional down-regulation in the context of the practical and emotional demands of caregiving.

Regarding predictors of university student carer outcomes, demographic variables as a block made a significant incremental contribution to anxiety symptoms, academic performance, and problematic involvement in videogaming, sexual activity, and social network use. At the coefficient level, sex and age emerged as significant predictors. However, the sex-related findings appear to reflect established population-level differences rather than caregiving-specific mechanisms: female sex was associated with higher anxiety symptoms and lower odds of problematic videogaming and sexual activity, in line with evidence that women typically report higher anxiety symptoms (Farhane-Medina et al., 2022; McLean et al., 2011), whereas problematic videogaming and compulsive sexual behavior are more consistently reported among men (Stevens et al., 2021; Bőthe et al., 2023; Kürbitz and Briken, 2021).

Educational-context variables as a block predicted depressive symptoms, academic performance, tobacco use, and cannabis use. However, no individual educational-context variables emerged as significant predictors. At the bivariate level most correlations between the educational-context variables and carer outcomes were non-significant, and the few significant correlations were small in magnitude, not exceeding 0.10. The educational variables measured in this study (degree programme, residential status, and enrolment status) appear not to be robust predictors of student carer mental health, addictive behavior and academic performance. This pattern is broadly consistent with student wellbeing research suggesting that structural or circumstantial student characteristics may be less informative than more proximal psychosocial and stress-related factors. For example, Brett et al. (2023) found that perceived stress, sense of coherence, loneliness, and resilience were the strongest predictors of university student wellbeing, whereas demographic and study-related variables did not remain significant in the final model.

Caregiving context variables, entered as a block, made a significant incremental contribution to depressive and anxiety symptoms and to problematic alcohol and tobacco use. In the final models, however, no care-recipient family status indicator survived FDR correction. Thus, there was no robust evidence that outcomes differed according to whether student carers supported a parent, sibling, grandparent, or another family member. By contrast, some care recipient health-condition indicators were associated with carer outcomes. Specifically, the presence of a family member with a mental health condition or intellectual disability was associated with higher depressive symptoms and higher odds of problematic sexual activity in the carer; the presence of a family member with physical illness or disability was associated with lower odds of problematic alcohol; whereas the presence of substance-related problems was associated with higher odds of problematic tobacco use. This pattern of associations between the care recipient's type of health condition and carer mental health is consistent with findings from other carer studies. For example, relative to caring for a family member with a physical illness, caring for a family member with mental illness has been associated with poorer mental health in university student carers (Crandall et al., 2014; van der Werf et al., 2019), young adult carers (Haugland et al., 2023; Pakenham and Cox, 2014), and carers recruited from community samples (Bremmers et al., 2025; Livingston et al., 1996). The present findings extend this literature by suggesting that care recipient health-condition indicators in the caregiving context may also be relevant to selected addictive-behavior outcomes among university student carers, outcomes that are rarely assessed in carer research.

Greater caregiving responsibilities predicted higher depressive and anxiety symptoms and more problematic food consumption and physical exercise after adjusting for demographic, educational, and caregiving context variables. Notably, controlling for the effects of the relevant background variables provides a relatively stringent test of the unique effects of caregiving responsibilities on student carer outcomes. These findings are consistent with cross-sectional and longitudinal studies that have found that higher caregiving responsibilities are a robust predictor of poorer mental health in young adult carers (Fleitas Alfonzo et al., 2022; Landi et al., 2022b) and adult carers (Kayaalp et al., 2021; Pakenham, 2012).

These findings have potential implications for how universities recognize and respond to the needs of student carers. Early identification may be important, and future research could examine the feasibility and acceptability of voluntary approaches to identifying student carers through existing institutional processes. Such approaches could include requests for information on the risk factors identified in this study, particularly the presence of a family member with mental illness or intellectual disability and caregiving responsibilities. Prospective and intervention research is also needed to determine which forms of support are effective. For example, future studies could evaluate time-limited academic accommodations during periods of increased caregiving demand, such as flexibility with assessment deadlines or course-load adjustments. Research could also examine the value of providing accessible information about academic, psychological, financial, and respite support, as well as stepped support pathways matched to individual student-carer needs. Potential approaches for evaluation include student-carer associations, peer-mentoring programmes, and transdiagnostic mental-health promotion interventions (e.g., Viskovich and Pakenham, 2020), with more personalized support, such as targeted case management, considered for students with greater caregiving-related needs or impairment.

Findings should be interpreted in the context of methodological limitations. First, convenience sampling from one university was used with an overrepresentation of females and as such the generalizability of findings are limited. Second, the study was cross-sectional leaving the temporal and causal direction among study variables ambiguous. Longitudinal research is required to provide insights into how variables are causally related. Third, the assessment of variables within each predictor domain (demographics, educational and caregiving) was not exhaustive. For example, according to the tripartite model of caregiving (Pakenham and Cox, 2015; Landi et al., 2022c), the construct includes caregiving tasks and experiences in addition to caregiving responsibilities. Additionally, there are likely to be other domains, such as coping resources, involved in shaping student carer outcomes. This study focused on mental health risk factors and the negative impacts of caregiving on student carers, future research, while advancing our knowledge of the causal relations among these variables, should also consider the positive outcomes of caregiving widely documented in other carer groups (e.g., Methi et al., 2024; Chiba et al., 2025; Pakenham and Cox, 2018) and mental health protective factors. Fourth, academic performance was self-assessed; future research should also include objective indicators, such as examination grades, GPA, and study progression. Fifth, because the survey was administered in Italian, eligibility required fluency in Italian; consequently, international students without sufficient Italian proficiency were excluded, which may limit the generalizability of the findings to this group and may have introduced language-related selection bias.

This study also has strengths which signal how results can contribute to the university student carer field. First, investigation into the incremental and simultaneous effects of potential risk factors on student carer mental health, addictive behaviors and academic performance provides insights into how predictors intersect to potentially shape university student carer outcomes. Second, this is the only published study to include a wide range of university student carer addictive behaviors as outcomes. Third, this is the first published study to employ a self-assessment measure of academic performance which has undergone rigorous development and testing. Fourth, the sample size is relatively large enabling the use of robust statistical analyses. Fifth, this is the first published study on university student carers in Italy, and in this regard contributes to our cross-cultural understanding. In summary, the present study provides a broad investigation of numerous potential predictors of a wide range of university student outcomes and identifies factors that may inform future public health research and intervention development.

### 4.1 Conclusions

Findings from this study replicate prior evidence showing that university student carers are at higher risk than non-carer peers for mental health problems. Results extend this body of research showing that student carers are also at higher risk for some addictive behaviors (cannabis use, shopping, videogaming, food consumption, and social network use) and poorer self-assessed academic performance. Additionally, findings highlight the incremental and simultaneous contributions of demographic, educational, and caregiving variables as risk factors for student carer mental health problems and addictive behaviors. Our inclusive modeling of multiple variables as predictors of student carer outcomes provides a foundation for further testing of a wider range of potential risk factors in future studies. The detection of mental health risk factors in the present study may help inform the identification of university student carers who may require additional support and priorities for future prevention research. Given that the increase in mental health problems among university students is an emerging global public health priority in the higher education sector, the identification of caregiving as a key mental health risk factor and the recognition of factors that can exacerbate the adverse impacts of caregiving in this population represents an important advancement in this field. Prospective, multicenter studies using more comprehensive assessments of caregiving and validated behavioral thresholds are needed to clarify these associations and establish their temporal and clinical significance, and to evaluate voluntary, needs-responsive support approaches aimed at reducing academic and psychological strain among students with substantial caregiving responsibilities.

## Data Availability

The raw data supporting the conclusions of this article will be made available by the authors, without undue reservation.
