# Supplementary material for: Mental health, academic performance, and addictive behaviors among university student carers
Source: Front Psychol. 2026 Oct 14;17:1971038. doi: 10.3389/fpsyg.2026.1971038 (PMC13713791; doi:10.3389/fpsyg.2026.1971038)
Supplement: Supplementary file 1 [file Supplementary_file_1.docx]

Supplementary Materials

# 1 Supplementary Table S1. Bivariate correlations between demographic, educational, and caregiving-related variables and mental health and academic performance outcomes in the carer subsample.

|  | Depressive symptoms (PHQ-9) | Anxiety symptoms  (GAD-7) | Academic performance |
| --- | --- | --- | --- |
| Sex (female) | .08* | .14*** | −.01 |
| Age 23–25 | −.02 | .02 | .04 |
| Age 26–35 | .06 | .04 | −.13** |
| Age > 35 | −.04 | −.06 | −.02 |
| Nationality (Italian) | −.05 | −.01 | .12** |
| Master’s degree | −.07 | .00 | .08* |
| Single-cycle degree | .08* | .04 | −.08* |
| PhD | −.05 | −.02 | −.04 |
| Commuter | −.01 | −.04 | .03 |
| Non-resident | .02 | .02 | .04 |
| Part-time student | .05 | .08* | −.07 |
| Working student | .03 | .03 | −.08* |
| Mental health/intellectual disability | .16*** | .16*** | −.06 |
| Physical illness or disability | −.09* | −.10** | .02 |
| Substance-related problems | .07 | .09* | .01 |
| Carer of a parent | .06 | .04 | −.07 |
| Carer of a sibling | .12** | .07 | −.04 |
| Carer of a grandparent | −.12** | −.08* | .09* |
| Carer of another family member | .01 | .04 | −.03 |
| Caregiving responsibilities | .20*** | .20*** | −.11** |

*Note.* N ranges from 651 (academic performance) to 755 (mental health outcomes) owing to pairwise deletion. Coefficients are Pearson correlations. Correlations between dummy-coded predictors and continuous outcomes correspond to point-biserial correlations, whereas correlations between caregiving responsibilities and continuous outcomes are standard Pearson correlations.

* *p* < .05, ** *p* < .01, *** *p* < .001 (two-tailed).

# 2  Supplementary Table S2. bivariate correlations between demographic, educational, and caregiving-related variables and substance and behavioral addiction outcomes in the carer subsample.

|  | Alcohol | Tobacco | Cannabis | Shopping | Videogaming | Food consumption | Sexual activity | Studying | Social network | Physical exercise |
| --- | --- | --- | --- | --- | --- | --- | --- | --- | --- | --- |
| Sex (female) | −.01 | −.01 | −.03 | .07* | −.21*** | .03 | −.42*** | .04 | .07 | −.08* |
| Age 23–25 | −.01 | −.04 | .03 | −.05 | −.02 | −.02 | .01 | −.01 | .00 | .02 |
| Age 26–35 | .01 | .03 | −.01 | .01 | .01 | .01 | −.07 | −.01 | −.07* | −.09* |
| Age > 35 | −.05 | −.04 | −.07 | .03 | −.07 | .00 | .00 | .01 | −.18*** | .04 |
| Nationality (Italian) | .01 | .05 | .03 | −.03 | −.04 | .02 | .02 | .02 | .04 | −.02 |
| Master’s degree | .00 | −.04 | .00 | −.02 | −.05 | .00 | −.04 | .00 | −.04 | .00 |
| Single-cycle degree | −.05 | −.05 | −.10** | .00 | .00 | .01 | −.06 | .06 | .01 | −.01 |
| PhD | −.04 | −.01 | .01 | −.05 | −.03 | −.08* | −.04 | −.02 | −.03 | .02 |
| Commuter | −.06 | −.06 | −.03 | −.05 | .03 | .03 | .03 | −.01 | − .00 | .02 |
| Non-resident | .07* | .08* | .04 | .01 | −.06 | .02 | −.01 | .00 | .01 | −.04 |
| Part-time student | .05 | .03 | .10** | .05 | .02 | .05 | .02 | −.03 | .06 | .02 |
| Working student | .02 | .07* | .02 | .01 | −.07 | .05 | .04 | −.02 | −.09* | .07 |
| Mental health/intellectual disability | .05 | .05 | .02 | .04 | .02 | .07* | .07* | .02 | .05 | .01 |
| Physical illness or disability | −.15*** | −.08* | −.08* | −.01 | .04 | −.01 | .00 | −.01 | −.02 | .02 |
| Substance-related problems | .14*** | .15*** | .10** | .07 | .00 | .07 | .01 | − .00 | .02 | −.01 |
| Carer of a parent | −.02 | .06 | .07* | .03 | .04 | .04 | .02 | −.10** | .06 | −.05 |
| Carer of a sibling | .02 | .02 | −.05 | −.01 | −.03 | .02 | .01 | .00 | −.04 | .05 |
| Carer of a grandparent | −.03 | −.08* | −.07 | −.03 | −.01 | −.04 | −.04 | .09* | −.01 | .02 |
| Carer of another family member | .01 | .00 | − .00 | .03 | −.02 | .04 | .02 | −.02 | −.09* | .00 |
| Caregiving responsibilities | .00 | .05 | .01 | .07 | .01 | .14*** | −.01 | .03 | −.02 | .08* |

*Note.* Each outcome is a binary indicator of problematic involvement, coded 0 = below cut-off and 1 = at or above cut-off. N ranges from 738 to 755 across outcomes owing to pairwise deletion. Coefficients are Pearson correlations. Correlations between dummy-coded predictors and binary outcomes correspond to phi coefficients, whereas correlations between caregiving responsibilities and binary outcomes correspond to point-biserial correlations.

* *p* < .05, ** *p* < .01, *** *p* < .001 (two-tailed).

# 3 Supplementary Table S3. Intercorrelations among demographic, educational, and caregiving-related variables in the carer subsample.

|  | **1** | **2** | **3** | **4** | **5** | **6** | **7** | **8** | **9** | **10** | **11** | **12** | **13** | **14** | **15** | **16** | **17** | **18** | **19** |
| --- | --- | --- | --- | --- | --- | --- | --- | --- | --- | --- | --- | --- | --- | --- | --- | --- | --- | --- | --- |
| 1. Sex (female) | — |  |  |  |  |  |  |  |  |  |  |  |  |  |  |  |  |  |  |
| 2. Age 23–25 | −.03 | — |  |  |  |  |  |  |  |  |  |  |  |  |  |  |  |  |  |
| 3. Age 26–35 | −.02 | −.39*** | — |  |  |  |  |  |  |  |  |  |  |  |  |  |  |  |  |
| 4. Age > 35 | −.02 | −.15*** | −.10** | — |  |  |  |  |  |  |  |  |  |  |  |  |  |  |  |
| 5. Nationality (Italian) | −.01 | .06 | −.09* | −.20*** | — |  |  |  |  |  |  |  |  |  |  |  |  |  |  |
| 6. Master’s degree | −.02 | .36*** | .09* | −.05 | .02 | — |  |  |  |  |  |  |  |  |  |  |  |  |  |
| 7. Single-cycle degree | .08* | .06 | −.08* | .04 | .05 | −.33*** | — |  |  |  |  |  |  |  |  |  |  |  |  |
| 8. PhD | −.04 | −.08* | .34*** | .05 | −.05 | −.20*** | −.12** | — |  |  |  |  |  |  |  |  |  |  |  |
| 9. Commuter | .00 | −.10** | −.02 | .01 | .05 | −.06 | −.06 | −.03 | — |  |  |  |  |  |  |  |  |  |  |
| 10. Non-resident | .06 | .13*** | −.07 | −.07 | .13*** | .08* | .12** | −.09* | −.71*** | — |  |  |  |  |  |  |  |  |  |
| 11. Part-time student | .06 | .01 | −.03 | −.02 | −.02 | .03 | −.06 | −.06 | .06 | −.08* | — |  |  |  |  |  |  |  |  |
| 12. Working student | .03 | −.04 | .18*** | .21*** | −.03 | .07 | −.06 | .10** | .08* | −.12** | −.12** | — |  |  |  |  |  |  |  |
| 13. Mental health/intellectual disability | .06 | .05 | −.01 | .02 | .05 | .03 | .06 | −.05 | −.05 | .05 | .02 | .07 | — |  |  |  |  |  |  |
| 14. Physical illness or disability | −.04 | −.03 | −.02 | −.05 | .02 | −.04 | −.02 | .05 | .09* | −.09* | −.07 | .00 | −.54*** | — |  |  |  |  |  |
| 15. Substance-related problems | .03 | .02 | .05 | −.03 | −.04 | −.02 | −.01 | −.02 | −.06 | .03 | .02 | .03 | −.01 | −.20*** | — |  |  |  |  |
| 16. Carer of a parent | .03 | .03 | .03 | −.08* | −.06 | −.02 | −.02 | −.03 | .05 | −.09* | −.01 | −.02 | −.08* | .11** | .10** | — |  |  |  |
| 17. Carer of a sibling | .01 | .03 | −.01 | −.09* | .07 | −.02 | .03 | −.02 | .01 | .06 | .02 | .02 | .20*** | −.14*** | −.01 | −.17*** | — |  |  |
| 18. Carer of a grandparent | −.01 | .01 | −.04 | −.11** | .11** | .02 | .04 | .01 | −.02 | .06 | .03 | −.04 | .00 | .17*** | −.12** | −.53*** | −.22*** | — |  |
| 19. Carer of another family member | −.03 | −.07 | .09* | .38*** | −.13*** | .02 | −.05 | .09** | −.05 | −.03 | −.02 | .19*** | .06 | −.11** | .07 | −.30*** | −.13*** | −.12** | — |
| 20. Caregiving responsibilities | .15*** | −.01 | .06 | .05 | .05 | −.04 | .08* | −.01 | .08* | −.08* | .02 | .14*** | .15*** | .01 | .02 | .14*** | .03 | −.03 | −.05 |

*Note.* Coefficients are Pearson correlations. Correlations between two dummy-coded predictors correspond to phi coefficients, whereas correlations between caregiving responsibilities and dummy-coded predictors correspond to point-biserial correlations. Correlations were computed in the carer subsample using pairwise deletion.

* *p* < .05, ** *p* < .01, *** *p* < .001 (two-tailed).
